# Elevated serum mtDNA in COVID-19 patients is linked to SARS-CoV-2 envelope protein targeting mitochondrial VDAC1, inducing apoptosis and mtDNA release

**DOI:** 10.1007/s10495-024-02025-5

**Published:** 2024-10-07

**Authors:** Anna Shteinfer-Kuzmine, Ankit Verma, Rut Bornshten, Eli Ben Chetrit, Ami Ben-Ya’acov, Hadas Pahima, Ethan Rubin, Yosef Mograbi, Eyal Shteyer, Varda Shoshan-Barmatz

**Affiliations:** 1 National Institute for Biotechnology in the Negev, Beer-Sheva, Israel; 2 Department of Life Sciences, Ben-Gurion University of the Negev, 84105 Beer-Sheva, Israel; 3 The Shraga Segal Dept. of Microbiology, Immunology and Genetics, Ben-Gurion University of the Negev, 84105 Beer-Sheva, Israel; 4 Infectious Diseases Unit, Shaare Zedek Medical Center, Hebrew University School of Medicine, Jerusalem, Israel; 5 Shaare Zedek Medical Center, The Juliet Keidan Institute of Paediatric Gastroenterology, Jerusalem, Israel; 6 Tel Aviv-Yafo, Israel

**Keywords:** Apoptosis, COVID-19, mtDNA, Mitochondria, VDAC1

## Abstract

Mitochondria dysfunction is implicated in cell death, inflammation, and autoimmunity. During viral infections, some viruses employ different strategies to disrupt mitochondria-dependent apoptosis, while others, including SARS-CoV-2, induce host cell apoptosis to facilitate replication and immune system modulation. Given mitochondrial DNAs (mtDNA) role as a pro-inflammatory damage-associated molecular pattern in inflammatory diseases, we examined its levels in the serum of COVID-19 patients and found it to be high relative to levels in healthy donors. Furthermore, comparison of serum protein profiles between healthy individuals and SARS-CoV-2-infected patients revealed unique bands in the COVID-19 patients. Using mass spectroscopy, we identified over 15 proteins, whose levels in the serum of COVID-19 patients were 4- to 780-fold higher. As mtDNA release from the mitochondria is mediated by the oligomeric form of the mitochondrial-gatekeeper—the voltage-dependent anion-selective channel 1 (VDAC1)—we investigated whether SARS-CoV-2 protein alters VDAC1 expression. Among the three selected SARS-CoV-2 proteins, small envelope (E), nucleocapsid (N), and accessory 3b proteins, the E-protein induced VDAC1 overexpression, VDAC1 oligomerization, cell death, and mtDNA release. Additionally, this protein led to mitochondrial dysfunction, as evidenced by increased mitochondrial ROS production and cytosolic Ca^2+^ levels. These findings suggest that SARS-CoV-2 E-protein induces mitochondrial dysfunction, apoptosis, and mtDNA release via VDAC1 modulation. mtDNA that accumulates in the blood activates the cGAS-STING pathway, triggering inflammatory cytokine and chemokine expression that contribute to the cytokine storm and tissue damage seen in cases of severe COVID-19.

**Supplementary Information:**

The online version contains supplementary material available at 10.1007/s10495-024-02025-5.

## Introduction

Coronaviruses (CoVs) are enveloped, single-stranded RNA viruses that have a wide range of natural hosts. Among the seven CoVs known to infect humans, only three—SARS-CoV-2, MERS-CoV, and SARS-CoV—have been associated with severe acute respiratory syndrome (SARS).

SARS-CoV-2 manifests across a broad spectrum of clinical presentations that range from mild respiratory symptoms to pneumonia. In severe cases, an exaggerated inflammatory response, often termed a “cytokine storm”, ensues, characterized by elevated levels of circulating tumor necrosis factor (TNF) and interleukin-6 (IL-6) [1]. This cytokine storm attracts mononuclear cells to the lungs [2], which contributes to tissue damage and multi-organ failure, and ultimately leads to death.

The SARS-CoV genome comprises two-thirds of the RNA that is located within the first open reading frame (ORF1a/b). This translates into two polyproteins, pp1a and pp1b, that give rise to 16 non-structural proteins. The remaining portion of the viral genome encodes several structural and accessory proteins: the spike (S) protein, a transmembrane glycoprotein that is present on the viral surface, cleaved by host-cell proteases, which after anchoring enters the host cells via angiotensin receptor-2 (ACE2): the matrix (M) protein responsible for nutrient transport; the small envelope (E) protein, an integral membrane protein; and nucleocapsid (N) protein, which forms the capsid surrounding the RNA genome [2]. In addition to these proteins, SARS-CoV encodes various accessory proteins, such as 3a and 3b proteins, which play roles in virus pathology. These accessory proteins interfere with the host innate immune response, and they contribute to mitochondria dysfunction and apoptosis [3, 4].

Viruses have evolved distinct strategies to evade biochemical and immunological defenses of the host, including the regulation of apoptosis, inflammation, and immune reactions. Some viruses induce apoptosis, either by eliminating uninfected immune cells or breaking down infected cells to facilitate viral spread [5]. Viruses inhibit or induce apoptosis in various tissues and in different ways [6]—some by regulating the expression of proteins from the Bcl-2 family (such as Bid, Bcl2) or by mimicking them [5, 7], and others either directly by viral proteins [6, 8] or indirectly by activating cellular mechanism-leading to apoptosis that enhance viral infection efficiency [9] often by directly targeting the mitochondria [10]. As an example, HIV-1 induces neuronal and immunological damage through mitochondria-mediated apoptosis by at least three distinct mechanisms [11]. Viruses also modulate host cell metabolism and physiology, which results in altered cellular functions [12].

The mitochondria control cellular homeostasis, metabolism, innate immunity, apoptosis, and more [13–15]. They play a key function in apoptosis with VDAC1, situated on the outer mitochondrial membrane (OMM), acting as a mitochondrial gatekeeper that controls both metabolic and apoptostic functions. VDAC1 modulates apoptosis by binding anti-apoptotic proteins and mediating the release of cytochrome c and other pro-apoptotic factors [16–18].

Upon induction of apoptosis by various inducers, such as Ca^2+^ or UV irradiation, VDAC1 expression levels rise, shifting the equilibrium towards an oligomeric state with a large pore, which allows the release of mitochondrial pro-apoptotic proteins [19]. Thus, the assambly of this new oligomeric VDAC1 channel is induced by diverse initiating cascades, stressors, and apoptotic triggers [16, 18, 20–22]. Notably, the channel also releases mitochondrial DNA (mtDNA) [23].

mtDNA shares many similarities with immunogenic bacterial DNA and is recognized as a pro-inflammatory damage-associated molecular pattern (DAMP) that contributes to the pathogenesis of various inflammatory diseases. mtDNA was demonstrated to be an inevitable factor during cGAS-STING activation in SARS-CoV-2 infection and an inhibitor of VDAC1-oligomerization that we developed [24], VBIT-4, which suppressed mtDNA release [23] protected against cGAS-STING activation in SARS-CoV-2 infection [25].

Numerous studies have illustrated the pivotal role of the mitochondria, particularly involving VDAC1, in the pathogenic mechanisms of various viruses. These viruses employ diverse strategies, such as inducing VDAC1 overexpression or directly interacting with it, thereby modulating mitochondrial function. Additionally, silencing VDAC1 expression has been shown to significantly reduce the expression of viral proteins [19, 26]. Examples of this are: (i) in the hepatitis B virus (HBV), HBx protein forms a complex with VDAC1 that impacts mitochondrial physiology [27]; (ii) expression of the hepatitis E virus (HEV) capsid protein Orf3 increases VDAC1 levels [28]; (iii) human immunodeficiency virus type 1 (HIV-1) protein R (Vpr) interacts with VDAC1, causing cell cycle arrest and apoptosis in infected T-lymphocytes [29]; (iv) downregulation of VDAC1 expression results in reduced expression of Dengue virus (DENV) proteins NS1, NS3, NS5, and DENVE [30]; (v) infectious bursal disease virus (IBDV) protein VP5 induces apoptosis by interacting with VDAC1, and silencing VDAC1 expression decreases the expression of VP1, VP2, and VP5 [31], and (vi) coronaviruses, including SARS-CoV-2, encode both pro-apoptotic and anti-apoptotic proteins, regulating apoptosis progression [32]. A distinct population of T cells in COVID-19 patients exhibits significant upregulation of VDAC1 and VDAC1-dependent apoptosis that can be inhibited by VBIT-4 [33].

Building on these findings, the current study examined the relationship between SARS-CoV-2-derived proteins and the mitochondrial protein VDAC1, focusing on apoptosis and mtDNA release. We investigated the role of VDAC1 in the pathogenesis of SARS-CoV-2, which manifests as COVID-19. Among the array of proteins encoded by SARS-CoV, we selected four that are associated with virus pathology and are connected to mitochondria dysfunction and apoptosis [2]. The envelope (E-) protein has been shown to translocate to the cell surface, altering the host-cell membrane’s permeability to facilitate viral entry, which promotes replication, proliferation, and inflammation cascade induction by SARS-CoV [34]. The N-protein induces apoptosis via the mitochondrial apoptotic pathway under starvation of serum, increasing ROS levels, which leads to the loss of membrane potential (ΔΨm) and induces apoptosis [35]. The SARS-CoV accessory 3a protein induces extensive cell death via mitochondrial pathways [3], and the SARS-CoV accessory 3b protein, proposed to contribute to SARS pathogenesis in humans, is reported to be localized to the nucleolus and mitochondria, inducing cell growth arrest [4, 36], apoptosis, and necrosis [37]. The 3a and 3b proteins modulate the host innate immune response to SARS-CoV infection by inhibiting interferon production and signaling [38].

Our investigation reveals that the cell expression of the E-protein of SARS-CoV-2 results in VDAC1 overexpression, oligomerization, and subsequent cell death. Given that VDAC1 oligomers are implicated in the release of mtDNA [23] and considering the protective effect of a VDAC1-oligomerization inhibitor VBIT-4 against cell death [16, 18, 20–22] and mtDNA release [23], as well as the cytokine storm induced by SARS-CoV-2 infection [25], we analyzed the presence of mtDNA in the serum of COVID-19 patients and searched for SARS-CoV-2 protein targeting VDAC1. In this paper, we present evidence of elevated levels of mtDNA and of specific proteins in the serum of COVID-19 patients. Additionally, we show that the E-protein, by inducing VDAC1 overexpression and oligomerization, allows the release of pro-apoptotic proteins and mtDNA, leading to cell death and to inflammation. These findings strongly imply the involvement of VDAC1 in the pathology of this virus.

## Materials and methods

### Materials

Propidium iodide (PI), Ponseau S, acridine orange, ethidium bromide, tris (hydroxymethyl)aminomethane, Tween-20, digitonin, and protease inhibitor cocktail were obtained from Sigma (St. Louis, MO). Phosphate-buffer saline (PBS), Dulbecco’s Modified Eagle’s Medium (DMEM), supplement fetal bovine serum (FBS), and penicillin–streptomycin were obtained from Gibco (Grand Island, NY). Dimethyl sulfoxide (DMSO) was purchased from MP Biomedicals (Solon, OH). Polyvalent JetPrime transfection reagent was from Polyplus (llkirch-Graffenstaden, France) and Prime Fect was from RJH Biosciences (Edmonton, Alberta, Canada). Ethylene glycol bis(succinimidyl succinate) (EGS), MitoSOX™ red mitochondrial superoxide indicator and Fluo-4 AM cell permeant were obtained from Thermo Fisher (Waltham, MA, US). Annexin V was purchased from Enzo Life Sciences (Farmingdale, NY, US). Hank’s Balanced Salt Solution (HBSS) was from Biological Industries (Beit-Haemek, Israel). Power SYBER Green Master Mix was obtained from Applied Biosystems (Foster City, CA).

### Subjects

Blood samples were collected from 51 hospitalized patients infected with COVID-19 and 24 healthy non-hospitalized controls without any infection, matched for age, from the COVID-19 biobank established at Shaare Zedek Medical Center (SZMC) in Jerusalem, Israel during the pandemic (March 6 to May 30, 2020). Clinical and demographic data, including age, gender, degree of oxygen support, severity of illness, and lab parameters were obtained from the computerized medical records or through interviews with study participants (see Table S1). The severity of COVID‐19 was evaluated based on the NIH guidelines (https://www-covid19treatmentguidelines-nih-gov.szmc.idm.oclc.org). Mild illness: Individuals with any of the various signs and symptoms of COVID-19 (e.g., fever, cough, sore throat, malaise, headache, muscle pain, nausea, vomiting, diarrhea, loss of taste and smell), but who do not have shortness of breath, dyspnea, or abnormal chest imaging. Moderate illness: Individuals who show evidence of lower respiratory disease during clinical assessment or imaging and who have an oxygen saturation measured by pulse oximetry (SpO_2_) ≥ 94% in room air at sea level. Severe illness: Individuals who have an SpO_2_ < 94% in room air at sea level. Critical illness: Individuals who have respiratory failure, septic shock, or multiple organ dysfunction. The study was conducted in accordance with the Helsinki guidelines and received approval from the SZMC Institutional Review Board.

### Cell culture and transfection

SHSY-5Y (CRL-2266, Human neuroblastoma, epithelial), A549 (CCL-185, human lung carcinoma, epithelial-like), and PC-3 (CRL1435, human prostate, adenocarcinoma) cell lines were obtained from the American Type Culture Collection (ATCC) (Manassas, VA). They were maintained using Dulbecco’s Modified Eagle’s Medium (DMEM), supplemented with 10% inactivated fetal bovine serum (FBS), and with 100 U/mL penicillin and 100 μg/mL streptomycin. Cells were grown at 37°C in 5% CO_2_ in a humidified incubator. Cell lines were routinely tested for mycoplasma contamination.

Cells were transiently transfected with 1 or 2 μg DNA for 24h, 48h, or 72h. The DNA included either an empty pcDNA3.1 plasmid or the pcDNA3.1 plasmid encoding for E-, N-, or 3b protein. Transfection was performed using JetPRIME transfection reagent (Polyplus, Illkirch-Graffenstaden, France) following the manufacturer’s instructions. The N, E, and 3b genes were cloned into a pcDNA3.1 vector with BamHI (GGATCC) and EcoRI (GAATTC) cloning sites and conferred ampicillin resistance (Synbio Technologies; NJ, USA).

### mtDNA isolation and analysis

Total serum DNA was extracted using a DNeasy Blood & Tissue Kit (Qiagen), according to the manufacturer’s instructions. mtDNA encoding for ND2, COX-III, D-Loop 1, and D-Loop 2 was analyzed using specific primers (Table S2) and quantified through quantitative real-time PCR (qPCR) with SYBR Green. Amplification of samples was conducted using a 7300 Real-Time PCR System (Applied Biosystems). For quantification of mtDNA released to the cytosol, cells were harvested 48h post transfection, pelleted, and re-suspended in 170 μl of buffer, containing 150 mM NaCl, 50 mM HEPES pH 7.4, and 25 µg/ml digitonin. Cells then were incubated for 10 min at room temperature onto a rotator, followed by centrifugation at 16,000 g at 4°C for 25 min. For q-RT-PCR, a 1:10 supernatant dilution was used to determine mtDNA using the specific primers listed in Table S2.

### Chemical cross-linking

Following the designated treatment, cells were collected and incubated in PBS at pH 8.3 (1 mg/ml) containing the cross-linking reagent EGS (100 μM) for 15 min at 30 °C. Samples (60 µg protein) were then subjected to SDS-PAGE, followed by immunoblotting using anti-VDAC1 antibodies. Quantitative analysis of immuno-reactive VDAC1 dimer, trimer, and multimer bands was performed using Image J software.

### Cell death analyses

Cell death was analyzed by propidium iodide (PI) staining (final concentration of 6.25 g/mL), followed by flow cytometry with an iCyt sy3200 Benchtop Cell Sorter/Analyzer (Sony Biotechnology Inc., San Jose, CA, USA) and analysis with EC800 software. Apoptosis was analyzed by PI and nnexin V-FITC staining, which was carried out according to the manufacturer’s instructions with minor modifications. After transfection, cells were harvested (1,500 g, 5 min), washed and re-suspended in 200 µl of binding buffer (10 mM Hepes/NaOH, pH 7.4, 140 mM NaCl, and 2.5 mM CaCl_2_). Annexin V-FITC/PI staining was performed, and the samples were analyzed by flow cytometry. At least 10,000 events were recorded, represented as dot plots. Cell death was also analyzed by acridine orange and ethidium bromide staining (100 g/ml) and light microscopy (LX2-KSP; Olympus).

### Intracellular Ca^2+^ level analysis

Fluo-4-AM was used to monitor changes in cytosolic Ca^2+^ levels. SHSY-5Y cells were harvested 48h post transfection, collected (1,500 × g for 5 min), washed with Hank’s Balanced Salt Solution (HBSS) supplemented with 1.8 mM CaCl_2_ (HBSS +), and incubated with 2 μM Fluo-4 in 200 μl HBSS( +) buffer for 30 min at 37°C in a light-protected environment. After removing the excess dye by washing with HBSS( +), the cellular free Ca^2+^ concentration was promptly measured using an iCyt sy3200 Benchtop Cell Sorter/Analyzer (Sony Biotechnology Inc., San Jose, CA). At least 10,000 events were recorded by the FL2 detector, represented as a histogram, and analyzed by EC800 software (Sony Biotechnology Inc., San Jose, CA). Positive cells showed a shift to an enhanced level of green fluorescence (FL2).

### Reactive oxygen species (ROS) level analysis

To assess mitochondrial ROS accumulation, SHSY-5Y cells were collected 48h post transfection and then treated with 5 μM MitoSOX™ Red, a mitochondrial superoxide indicator for live-cell imaging, for 10 min at 37°C. Fluorescence intensity was measured using flow cytometry (iCyt, Sony Biotechnology, San Jose, CA). Analysis was performed as in the intracellular Ca^2+^ level analysis above.

### Gel electrophoresis and immunoblotting

Cells were lysed using lysis buffer (50 mM Tris–HCl, pH 7.5, 150 mM NaCl, 1 mM EDTA, 1.5 mM MgCl_2_, 10% glycerol, 1% Triton X-100), supplemented with a protease inhibitor cocktail for 15 min on ice. The lysates were subsequently centrifuged at 12,000xg (10 min at 4°C), and protein concentration was determined using a Lowry assay.

Protein aliquots (10–20 μg) were subjected to SDS-PAGE, and then were electro-transferred onto nitrocellulose membranes for immunostaining. The membranes were incubated with a blocking solution containing 5% non-fat dry milk and 0.1% Tween-20 in Tris-buffered saline (TBST), followed by incubation with primary antibodies. Subsequently, membranes were incubated with HRP-conjugated anti-mouse or anti-rabbit IgG as secondary antibodies. HRP activity was detected using an enhanced chemiluminescent substrate (Advantsa, San Jose, CA). Band intensity was quantified using Image J software or FUSION-FX (Vilber Lourmat, France).

### Purified VDAC1 and SARS-CoV-2 proteins

Purified SARS-CoV-2 nucleocapsid N-protein (N; S014660-05) and the envelope E-protein (MBS8309649) were obtained from Synbio Technologies (NJ, USA) or from MyBioSource (San Diego, CA). VDAC1 protein was purified from rat liver mitochondria following the protocol previously described [39].

### Micro-scale thermophoresis (MST) assay

MST analysis was performed using a NanoTemper Monolith NT.115 apparatus, as described previously [24, 40]. Briefly, purified VDAC1 or purified MAVS proteins were fluorescently labeled using a NanoTemper Protein labeling kit BLUE (L001, NanoTemper Technologies, GmbH). A constant protein concentration (138 nM for VDAC1 or 105 nM for MAVS) was incubated with different concentrations of the tested protein in an MST binding buffer (20mM Tris–HCl, 200mM NaCl, pH = 8) for 30 min at 37°C in the dark. Subsequently, 3–5 µl of the samples were loaded into a glass capillary (Monolith NT Capillaries), and a thermophoresis analysis was performed (LED 20%, IR laser 80%).

### Liquid chromatography-high-resolution mass spectrometry (LC-HR-MS) and proteomics analysis

Protein samples were subjected to SDS-PAGE followed by Coomassie staining and protein bands of ~ 50, 19, and 14 kDa were excised and subjected to in-gel digestion using trypsin, following the manufacturer’s protocol (Promega). Peptides were then extracted from the gel and loaded onto a LC-HR-MS.

LC/MS analysis was performed using an Eksigent nano‐HPLC (model nanoLC-2D, Netherlands) connected to an LTQ Orbitrap XL ETD (Thermo Fisher Scientific, Germany & USA). Peptide separation was achieved using a reverse-phase C-18 column [Acclaim PepMap, 75 ID (µm) 15 cm long: (Thermo Fisher Scientific)]. Peptides were eluted with a 70‐min linear gradient, starting with 100% buffer A (5% acetonitrile, 0.1% formic acid) to 80% buffer B (80% acetonitrile, 0.1% formic acid) at a flow rate of 300 nl/min. Full scans were acquired at 60,000 resolutions in the Orbitrap analyzer, followed by CID MS/MS analysis of the five most abundant peaks in the data-dependent mode. Fragmentation and detection of fragments were performed in the linear ion trap, with a minimum signal trigger threshold set at 500. The maximum ion fill-time settings were 500 ms for the high-resolution full scan in the Orbitrap analyzer and 200 ms for MS/MS analysis in the ion trap. The AGC settings were 5X10^5^ and 1X10^4^ for the Orbitrap and linear ion trap analyzers, respectively.

Proteins were identified and validated using SEQUEST search algorithms against the human protein sequences (NCBI and UniprotKB/SwissProt proteome database collection) operated under Proteome Discoverer 2.4 software (Thermo Fisher Scientific). Mass tolerances for precursors and fragmentations were set to 10 ppm and 0.8 Da, respectively. Only proteins containing at least two peptides of high confidence (Xcore 2 or 2.5 for doubly- or triply-charged species, respectively, or more) were selected.

An LC-HR-MS and proteomics analysis were carried out at the de Botton Institute for Protein Profiling, G-INCPM at the Weizmann Institute of Science in Rehovot, Israel, with an additional analysis performed at the Ilse Katz Institute for Nanoscale Science & Technology at Ben-Gurion University in Beer Sheva.

### Machine learning and statistics

For machine-learning tasks, models were built using the Python sklearn package (version 1.4.2), with or without dimension reduction techniques such as PCA, truncatedSVD, and sparsePCA algorithms. The models were trained using the fit function of the decision tree classifier object in sklearn. Validation was performed using a leave-one-out cross-validation approach. Unless otherwise stated, a significance cutoff of α = 0.05 was applied.

The data from cells in culture are shown as the mean ± SEM of at least three independent experiments. Significance of differences was calculated by a two-tailed Student’s t-test using the T-Test function provided by Microsoft Excel. Similarly, statistical significance between healthy and COVID-12 infected individuals was determined using Student’s t-test, conducted with the T-Test function in Microsoft Excel. Statistical significance is reported at p ≤ 0.05 (*), p ≤ 0.01 (**), or p ≤ 0.001 (***).

## Results

Given that certain viruses cause dysfunction of mitochondria and modulate VDAC1 expression and function [19, 26–32, 41], leading to cell death, inflammation, and autoimmune responses, we investigated the involvement of the mitochondria and VDAC1 in the pathology of SARS-CoV-2. Since mtDNA acts as a pro-inflammatory agent, we analyzed its levels in the serum of COVID-19 patients. In addition, we compared serum protein profiles of healthy and SARS-CoV-2-infected patients. Finally, we tested the effects of selected COVID-19 expression proteins on mitochondrial dysfunction, mtDNA release, and apoptosis induction.

### Patient characterization

During the pandemic period (March 6–April 7, 2020), blood samples were collected from 51 hospitalized patients infected with COVID-19. Onset of symptoms had occurred within 10 days prior to admission in 47/51 patients. Of the patients, 23 (45%) were female, with a mean age of 62.6 ± 17.7. Twenty-four healthy subjects served as controls: 12 (50%) were female, and mean age was 57 ± 14.6. (*p* = 0.18).

Of the COVID-19 patients, 17 were mildly ill, 10 had moderate disease, 21 had severe disease, and three were critically ill. Age above 65 was associated with severe or critical disease (OR 4.9, 95% CI 1.45–16.5, *p* = 0.008), as well as mean CRP level (OR 1.2 95% CI 1.08–1.4, *p* = 0001). A non-significant trend towards higher ferritin and d-dimer levels was observed among severely ill patients.

### Mitochondrial DNA (mtDNA) elevated in serum of COVID-19-infected patients

**Fig. 1:**
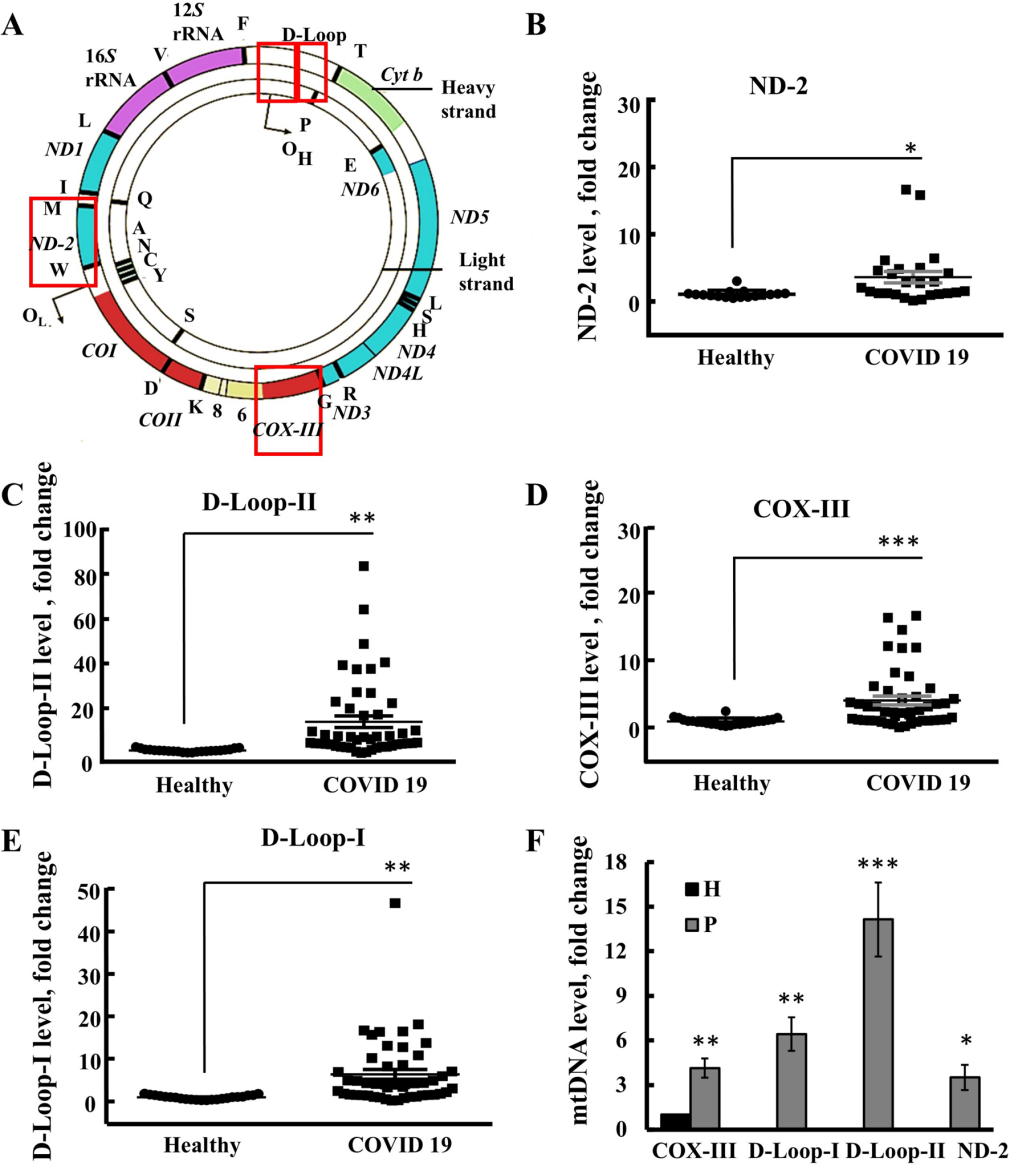
High levels of mtDNA found in serum of COVID-19 patients, relative to healthy donors. **A** The human mtDNA genome with the selected genes for analysis are marked in the square. The qPCR expression of mtDNA, **B** ND-2, **C** D-Loop-II, **D** COX-III, and **E** D-Loop-I in healthy (n = 24) and COVID-19 (n = 51) subjects. The fold change of mtDNA in the COVID-19 patients was higher than in healthy controls. Each dot represents an individual. **F** Summary of mtDNA for the indicated gene present in serum of the patients presented as a fold change relative to the levels in healthy donors. Results represent the means ± SEM, *p ≤ 0.05; **p ≤ 0.01; ***p ≤ 0.001

As SARS-CoV-2 induces COVID-19 in humans, and is linked to the activation of the cGAS-STING pathway triggered by mtDNA [25], we analyzed mtDNA levels in the blood of SARS-CoV-2-infected patients (Fig. 1). Intact mtDNA is large (16–17 kb) and anchored to the mitochondrial inner membrane (MIM) within nucleoid complexes [42]. However, as we previously demonstrated [23], short and free intra-mtDNA fragments (fmtDNA) are released from the mitochondria through the VDAC1 oligomer mega channel. The fmtDNA was isolated from heated serum samples of both healthy individuals and COVID-19 patients, and their quantities were determined using RT-qPCR specific primers (Table S2), targeting three genes and one non-coding sequence: cytochrome c oxidase subunit III (COX-III), NADH dehydrogenase 2 (ND-2), and the DNA displacement loop (D-Loop-I and D-Loop-II) (Fig. 1A). The D-Loop region, situated within the major non-coding region (NCR) of many mitochondrial genomes, is characterized by a triple-stranded structure formed by the stable integration of a third short DNA strand, known as 7S DNA. This region harbors crucial elements for transcription and replication processes [43].

High levels of mtDNA-derived fragments were found in the serum of SARS-CoV-2-infected patients compared to healthy donors (Fig. 1B–E). Specifically, while the average levels of COX-III, ND-2, and D-Loop-I were 4–sixfold higher relative to their levels in serum from healthy donors, the levels of D-Loop-II were an average of 13-fold higher (Fig. 1F). These results suggest higher production of mtDNA-D-Loop-II, indicating that the entire mtDNA molecule is not being released. This is consistent with previous findings that the sequences corresponding to a specific region within the D-Loop of the mitochondrial genome are overrepresented in the mtDNA pool [23].

We investigated whether the presence of mtDNA in sera could serve as an indicator of disease progression and/or severity. Multiple machine-learning analyses were conducted; however, the mtDNA sera levels did not emerge as a significant predictor of disease severity (Table S3).

Additionally, incorporating clinical data such as blood test results (e.g., white blood cells WBC), levels of proteins like D-dimer, ferritin, or CRP, or information about underlying conditions (e.g., hypertension) did not significantly improve prediction of disease severity.

As discussed below, the presence of plasma mtDNA exhibits a time-dependent pattern, with a peak observed at 24h post-disease onset [44]. This temporal variation may account for why our study failed to predict disease severity based on mtDNA levels, as the blood samples were collected at various time points following disease identification.

### Comparison of serum protein profiles of healthy and SARS-CoV-2-infected patients

**Fig. 2:**
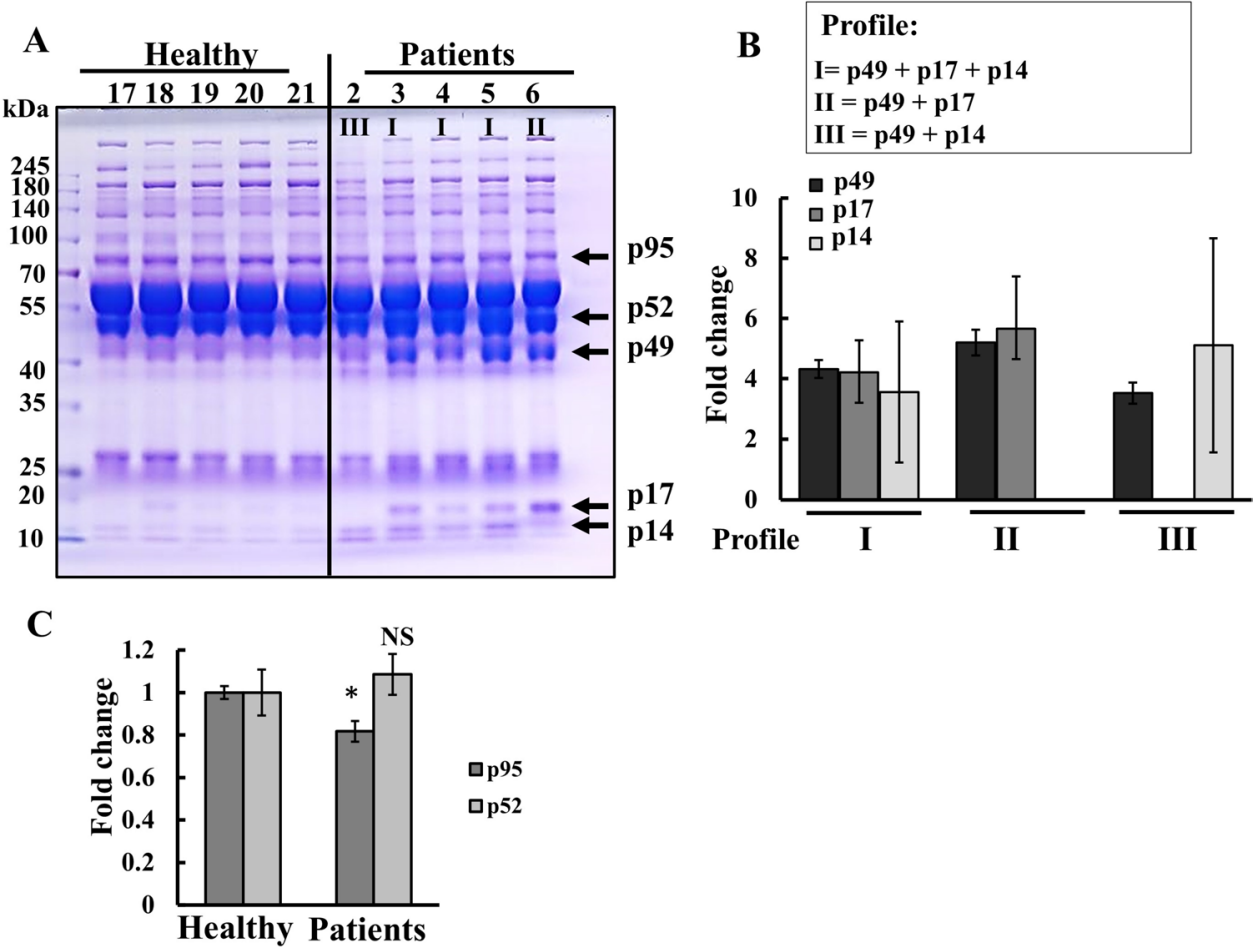
Serum of COVID-19 patients contains high levels of selected proteins. **A** Serum samples (30 μg protein) from patients and healthy donors were subjected to SDS-PAGE, followed by Coomassie blue staining. The protein bands found mainly in the COVID-19 samples are indicated and labeled as p95, p52, p49, p17, and p14, according to their estimated molecular mass. **B** Three categories of patients were defined: Protein profiles I, II, III, presenting p49 + p17 + p14, p49 + p17, and p49 + p14, respectively, are presented as the fold change (FC) relative to their levels in healthy donors. **C** Quantitative analysis of p52 and p95 levels in serum samples from healthy and COVID-19 samples. The apparent reduction in p95 levels in patients seems to be due to the presence of additional protein bands in the samples, which leads to a lower relative amount of the protein

Next, we conducted a comparison of protein profiles in serum samples obtained from healthy individuals and patients infected with SARS-CoV-2 using SDS-poly acryl-amide gel electrophoresis (SDS-PAGE) (Fig. 2A). We identified three protein bands, defined according to their apparent molecular mass as p49, p17, and p14, that were present in the serum from SARS-CoV-2-infected patients, but absent in that of the healthy group. We identified three distinct protein patterns and consequently defined three patient categories based on these profiles: **Profile I**: p49, p17, and p14 are present; **Profile II**: p49 and p17 are present, while p14 is undetectable; **Profile III**: p49 and p14 are present, while p17 is undetectable. These are presented as the fold change (FC) relative to their levels in healthy donors (Fig. 2B).

For comparison, the levels of two other proteins, p95 and p52, were analyzed and found to be similar in the sera from both COVID-19 and healthy donors (Fig. 2C).

A summary of the results of fmtDNA and differential present protein levels in serum obtained from COVID-19 patients and their diseases state is presented in Table 1.

**Table 1:** Summary of the results of the levels of mtDNA and differential present proteins in serum obtained from COVID-19 patients

		Level relative to healthy donors						
Patient no	Disease state	fmtDNA			Category	p49	p17	p14
		D-Loop-I	D-Loop-II	COX-III	Protein level FC			
P1	Severe	8.46	1.33	0.60	I	6.20	7.36	4.48
P2	Moderate	3.19	3.43	4.38	I	4.24	4.08	8.64
P3	Mild	14.46	18.28	2.67	I	8.50	7.69	5.59
P4	Moderate	3.81	6.26	3.38	I	6.59	5.92	4.29
P5	Severe	1.52	1.57	0.74	I	8.64	8.22	5.47
P6	Severe	1.71	3.15	1.61	II	7.61	11.75	-
P7	Severe	1.64	8.94	1.31	II	6.64	5.85	-
P8	Mild	1.85	3.76	1.97	II	5.30	5.41	-
P9	Moderate	4.10	4.71	3.49	I	5.70	2.97	9.28
P10	Severe	4.47	10.82	2.07	I	4.56	6.33	2.00
P11	Mild	3.74	4.66	2.30	III	5.86	-	8.66
P12	Mild	3.35	3.35	5.23	I	3.94	2.44	4.63
P13	Severe	ND	ND	ND	I	5.64	7.19	4.98
P14	Mild	1.68	2.99	1.14	I	4.60	4.55	4.48
P15	Severe (HFNC)	4.50	7.33	3.71	I	5.97	4.70	3.89
P16	Moderate	1.57	8.76	1.28	I	5.12	4.25	3.60
P17	Severe	1.90	8.42	1.16	I	4.32	3.19	6.34
P18	Moderate	1.62	31.37	3.27	I	7.29	6.78	2.69
P19	Moderate	0.93	3.64	1.50	II	3.89	4.64	2.69
P20	Critical	6	4.8	3.3		ND	ND	ND
P21	Critical	4.41	1.34	3.63	I	4.03	2.85	4.30
P22	Severe	1.51	2.91	1.26	I	4.20	2.42	2.83
P24	Mild	2.07	2.06	2.49	I	3.95	2.68	3.95
P25	Mild	1.99	2.75	1.21	I	3.80	4.88	2.39
P26	Severe	46.5	37.72	14.73		ND	ND	ND
P27	Mild	11.78	16.57	6.88	I	2.25	2.08	1.66
P28	Mild	15.03	37.40	5.69	I	1.72	2.98	4.55
P29	Severe	13.74	29.55	8.68	I	3.92	3.67	4.52
P30	Mild	7.99	6.08	3.07	I	2.21	1.81	3.22
P31	Severe	9.97	3.67	5.73	I	3.25	3.59	4.64
P32	Mild	0.15	0.17	1.13	I	3.54	5.38	4.29
P33	Severe	3.42	6.38	16.11	I	4.04	2.66	1.79
P34	Severe	3.69	3.25	17.10	I	3.76	4.59	1.54
P35	Mild	0.89	ND	0.79		ND	ND	ND
P36	Mild	5.50	3.91	0.93	I	1.69	1.52	1.31
P37	Moderate	3.61	2.83	1.30	I	2.92	1.45	0.72
P38	Mild	4.44	14.96	13.25	I	2.50	1.92	1.56
P39	Mild	13.8	17.4	2.4		ND	ND	ND
P40	Mild	3.15	48.8	12.3		ND	ND	ND
P41	Moderate	15.7	6.03	0.75		ND	ND	ND
P42	Severe	7.13	3.4	0.66	III	1.19	-	1.56
P43	Severe	12.71	6.05	1.44	I	1.19	2.38	1.69
P44	Moderate	17.55	76.58	11.18	I	3.73	12.98	1.71
P45	Severe	1.2	64.3	6.04		ND	ND	ND
P46	Severe	4.14	19.11	2.93	I	3.73	3.56	0.53
P47	Critical	1.7	7.7	3.4		ND	ND	ND
P48	Severe	0.6	8.3	1.36		ND	ND	ND
P49	Severe	0.02	0.04	ND		ND	ND	ND
P50	Mild	2.5	27.3	1.01		ND	ND	ND
P51	Moderate	13.92	23.59	3.98	I	4.51	5.04	2.63
P53	Severe	2.73	3.18	2.58	I	2.61	1.00	1.77

The COVID-19 patients, their disease state, levels of the three mtDNA-derived genes, and proteins present in their serum are presented. Also presented are the protein bands (p49, p17, p14) that were highly enriched in the serum of these patients

Three categories of patients were defined: I, those showing all three protein bands; II, those showing bands p49 and p17, and III, those showing p49 and p14, and their amount relative to healthy donors presented as a fold change (FC)

*ND* not determined

These proteins were also subjected to multiple machine-learning analyses to test whether they could serve as an indicator of disease progression or severity. However, their predictive value, alone or in combination of mtDNA levels, was not statistically significant.

To identify these proteins, the respective bands were excised from the gel and subjected to LC–MS/MS analysis in two different proteomics centers. We selected the LC–MS/MS-identified proteins exhibiting over 18% coverage, finding that all the identified peptides were unique to the corresponding protein. These results are summarized in Table 2, showing that the FC in protein levels between patients and healthy donors ranged from a 4- to 780-fold increase, also the number of the identified peptide unique to each identified protein, the percentage of the protein sequence covered by the identified peptides. The identified peptide sequences are located within the protein sequence (Fig. S1). The functions of these proteins and their possible association with COVID-19 are presented below and extended in the Supplementary Materials.

**Table 2:** Proteins identified by proteomics analysis of protein bands p49, p17, p14 obtained from COVID-19 patients

Protein	UniProt ID accession	Protein names	No of peptides	No of unique peptides	Fold change P/H	Sequence coverage (%)	MW kDa	Score
p49	P00738	Haptoglobin, isoform 1	21	21	Unique to patient	40	45.2	230.9
	P07358	Complement component C8 beta chain	10	10	273.9	19.8	63.5	66.6
	P01011	Alpha-1-antichymo-trypsin, His-pro-less	10	10	22.6	26.5	47.65	80.9
	P02768	Albumin	32	29	Unique to patient	59	69.3	59.1
	O43866	CD-5 antigen-like	15	15	Unique to patient	44	47.3	115.5
p17	P02741	C-reactive protein	7	7	290.3	17.9	25.03	55.6
	P55056	Apolipoprotein A-1	15	15	Unique to patient	56	30.8	30.3
	P01009	Alpha-1-anti-trypsin	13	13	26.0	25.6	46.73	135.7
	P01591	Immunoglobulin J chain	3	3	21.3	18.2	18.09	19.5
	P02753	Retinoic acid receptor protein	12	12	Unique to patient	63	23	21
	Q99969	Retinol-binding protein 4	4	4	Unique to patient	18.6	18.6	7.5
p14	P02766	Transthyretin	15	15	Unique to patient	78	15.9	60.6
	P35542	Serum amyloid A-4	9	9	3.9	55	12.8	60.2
	P55056	Apolipoprotein C-IV	6	6	Unique to patient	39	14.5	21.3
	P01834	Immunoglobulin kappa constant	10	10	Unique to patient	91	11.8	273

Protein bands p49, p17, and p14 were cut from the gel and subjected to trypsin digestion and LC-HR MS/MS analysis, performed as described in the Materials and Methods section

The protein band, uniport ID/accession number, protein name, number of peptides identified, and of these, the unique peptides, and the expression level in patients (P) relative to that in healthy donors (H) is presented as the fold of change, % of sequence coverage, molecular mass of the peptides and the scores are indicated

The p49 protein band was found to consist of several proteins including:

(1) **Haptoglobin**, which binds to hemoglobin, forming the haptoglobin–hemoglobin complex that is subsequently cleared by the reticuloendothelial system [45]; (2) **complement component C8**, a beta chain that is a constituent of the membrane attack complex (MAC) involved in mediating cell lysis and, thus, affects cell membrane integrity [46]; (3) **alpha-1-antichymotrypsin** (AACT), a circulating serine protease inhibitor derived from the liver; (4) **serum albumin,** which performs various essential functions such as maintaining plasma oncotic pressure and blood pH. It also acts as a carrier protein for steroids, hemin, fatty acids, hormones, amino acids, drugs, nutrients, and metal ions [47]; and (5) **CD5 antigen-like (CD5L)**, which is primarily secreted by macrophages in lymphoid tissues during inflammatory responses [48].

Within the p17 protein band, several proteins were identified, including:

(1) **C-reactive protein (CRP**), known for its rapid elevation in response to inflammation and infection [49, 50]; (2) **apolipoprotein A-1 (ApoA1),** a primary protein constituent of high-density lipoprotein (HDL) particles; (3) **alpha-1-anti-trypsin (AAT)**, a serine protease inhibitor that protects the lungs from neutrophil elastase which can damage lung tissue [51]; (4) **immunoglobulin Joining chain** (J chain), which is essential for the formation and stabilization of polymeric Ig structures [52]; (5) **retinoic acid receptor responder 2 (CRABP-II)**, a soluble cytosolic protein belonging to the family of intracellular lipid-binding proteins. It transports retinoic acid (RA), thereby enhancing its transcriptional activity [53]; and (6) **retinol-binding protein 4 (RBP4**), a transporter protein responsible for carrying retinol from liver stores to peripheral tissues [54]. Upon association with transthyretin (TTR), the retinol/RBP4/TTR complex is released into the bloodstream to deliver retinol to tissues [54].

The p14 protein band was found to contain several proteins:

(1) **Transthyretin** (TTR or TBPA), a tetrameric transporter of the thyroid hormone thyroxine and the retinol-binding protein (RBP) bound to retinol [55]; (2) **serum amyloid A4** (SAA4), an apolipoprotein found in high-density lipoproteins. Different isoforms of SAA are expressed constitutively at different levels or in response to inflammatory stimuli [56]; (3) **apolipoprotein C-IV** (APOC4), a lipid-binding protein with a role in lipoprotein metabolism [57]; and (4) **immunoglobulin kappa constant** (IGKC), representing one of the immunoglobulin (Ig) isotypes.

Finally, among the proteins exhibiting increased expression levels are those associated with immune response and inflammation. Notably, three of the identified proteins—CRABP-II, RBP4, and transthyretin—are involved in retinoid signaling, thus, influencing the regulation of Type I interferon synthesis and potentially contributing to excessive inflammation.

### Effects of the expression of the three SARS-CoV-2 encoded proteins on mitochondria-mediated apoptosis, VDAC1 expression levels, and VDAC1 oligomeric state

**Fig. 3:**
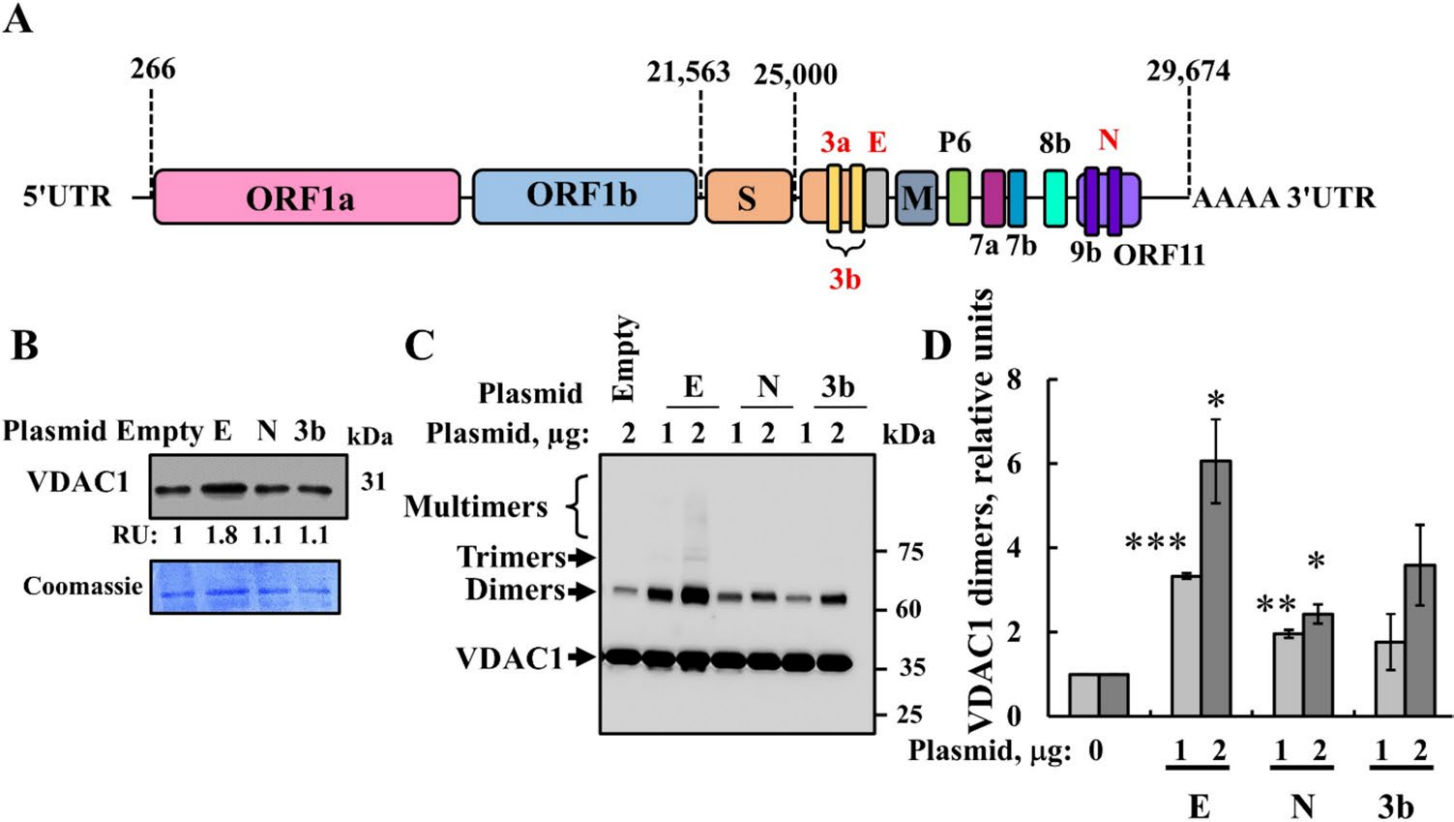
SHSY-5Y cell expression of SARS-CoV-2 E-protein induces VDAC1 overexpression and oligomerization. **A** (The SARS-CoV-2 genome contains the replicase gene, which encodes two open reading frames: ORF1a and ORF1b (two-thirds). The other genome encodes several ORFs including the spike glycoprotein (S), membrane (M), envelope (E), and nucleocapsid (N). ORFs encode several accessory proteins with their functions less well defined. ORFs 1a and 1b translated to produce the replicase pp1a and pp1ab polyproteins b [58]. **B** SHSY-5Y cells were transfected with 2μg pcDNA3.1 plasmid (empty) or encoding for proteins E, N, or 3b, and 48h post transfection, cells were harvested, lysed, and subjected to SDS-PAGE and immunoblotting using anti-VDAC1 antibodies. VDAC1 expression levels are presented in relative units (RUs), relative to their levels in the empty plasmid-transfected cells and are shown below the immunoblot. Coomassie blue stained of the blot is shown as a loading control. **C**, **D** SHSY-5Y cells were transfected with 1or 2 μg of the indicated plasmid (empty, E, N, or 3b) and 48h post transfection, cells were harvested and subjected to cross-linking using EGS (100 μM, 1 mg protein/ml) to monitor VDAC1 oligomerization as revealed by immunoblotting (**C**), and VDAC1 dimers were quantified using ImageJ software (**D**)

**Fig. 4:**
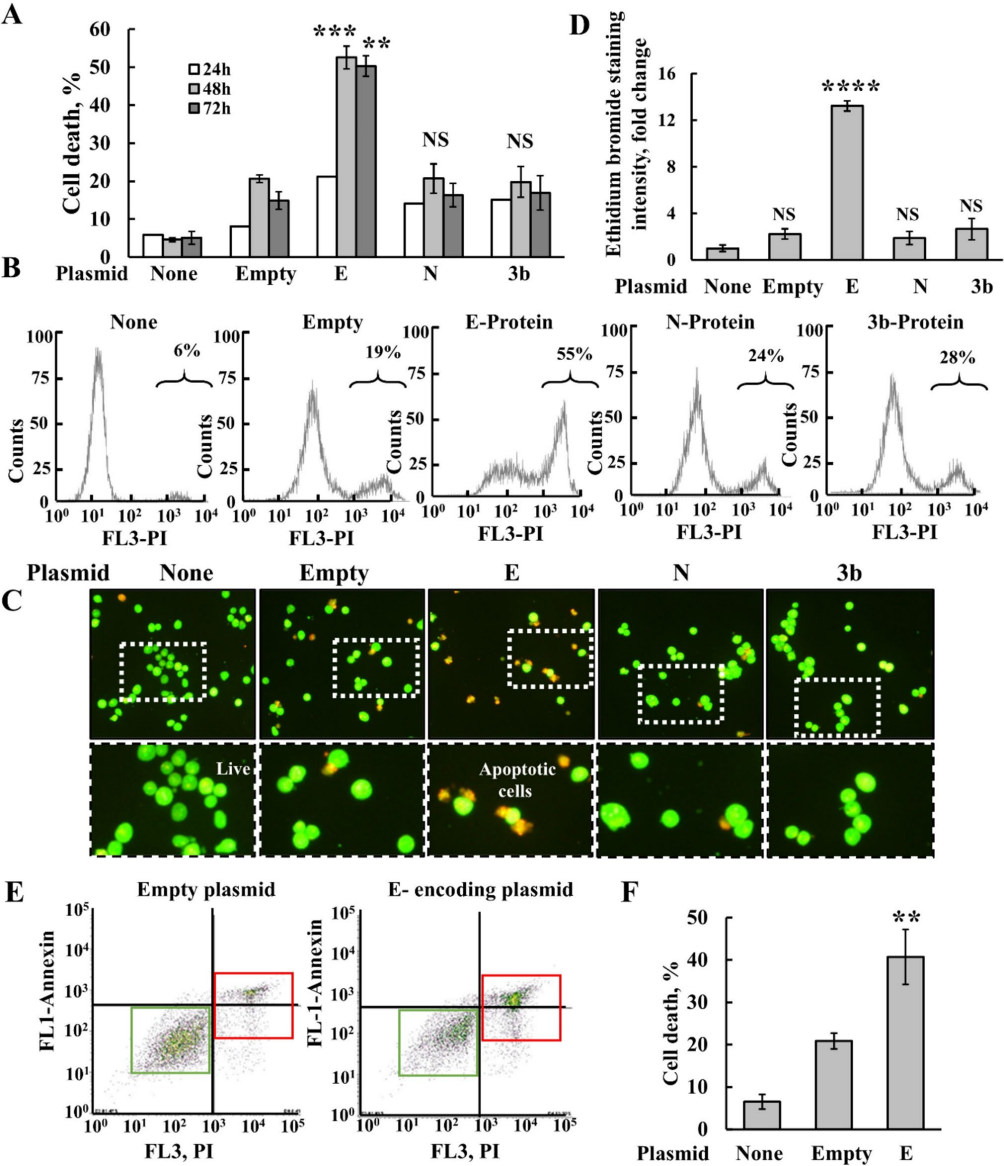
SHSY-5Y cell expression of SARS-CoV-2 E-protein induces cell death. **A**, **B** SHSY-5Y cells were transfected with 2 μg of pcDNA3.1 plasmid, empty, or encoding for E-, N-, or 3b proteins. 24h, 48h, or 72h post transfection, cells were harvested and analyzed for cell death using PI staining and flow cytometry. Quantitative analysis (**A**) and representative FACS histograms for the expressed viral proteins (**B**) are shown. Results represent the means ± SEM (n = 3) *p ≤ 0.05; **p ≤ 0.01; **p ≤ 0.001; *NS* non-specific. **C**, **D** Cells were transfected with 2 μg of pcDNA3.1 (empty) plasmid or encoding for E-, N-, or 3b proteins, and 48h post transfection, they were analyzed for cell death by staining with acridine orange and ethidium bromide, visualized with a microscope (LX2-KSP; Olympus). Representative images, captured by a CCD camera are shown, with the labeled squares pointing to the areas enlarged below (**C**), and quantification of ethidium bromide staining intensity (**D**). **E**, **F** SHSY-5Y cells were transfected with 2 μg of pcDNA3.1 plasmid (empty) or encoding for E-protein, and 48h post transfection, cells were subjected to PI/annexin V-FITC staining and flow cytometry analysis. Green and red boxes present live cells and dead cells, respectively. **F** Quantitative analysis of three independent experiments as shown in **E**. Results represent the means ± SEM (n = 3), **p ≤ 0.01; ****p ≤ 0.0001; NS = non-specific

Next, we selected SARS-CoV-2 proteins known to potentially induce mitochondrial dysfunction and apoptosis [3, 4]—the small envelope E- and nucleocapsid N-, and 3a- and 3b- proteins (Fig. 3A)—and tested their cell death induction and VDACI involvement. While the plasmid encoding 3a could not be successfully produced in bacteria, plasmids encoding for the E- and N- and 3b- proteins were successfully generated. Cells were transfected with E, N, 3b, or empty plasmid and, subsequently, assessed for apoptosis induction, VDAC1 expression levels, and VDAC1 oligomerization (Figs. 3B–D and 4). E-protein expression, but not N- and 3b, led to an increase in VDAC1 expression levels (Fig. 3B).

Next, VDAC1 oligomerization was assessed through chemical cross-linking with EGS and Western blotting using anti-VDAC1 antibodies. Several anti-VDAC1 antibody-labeled protein bands corresponding to VDAC1 dimers, trimers, tetramers, and higher-order multimers were detected in cells transfected to express E-, but not N-, or 3b protein (Fig. 3C, D).

Subsequently, we examined the impact of cells transfected with plasmids encoding E-, N-, and 3b proteins, and an empty plasmid on the induction of apoptotic cell death, as analyzed using several methods. In SHSY-5Y cells, only the E-protein induced apoptotic cell death, observed at 48 and 72h post transfection, as analyzed by propidium iodide (PI) and FACS analysis (Fig. 4A, B), aligning with its highest induction of VDAC1 expression and oligomerization (Fig. 3B–D).

To mitigate cell death caused by the empty plasmid, various transfection agents such as Prime Fect (RJH Biosciences; Edmonton, Alberta, Canada) and Jet Prime (Polyplus; Illkirch-Graffenstaden, France) were tested. However, cell transfection with the empty plasmid still resulted in some degree of cell death with all the transfection reagents used (data not shown).

Apoptosis induced by the expression of the three plasmids encoding for E-, but not N- or 3b protein, was also analyzed by acridine orange and ethidium bromide staining (Fig. 4C, D), showing that the expression of E-protein, but no other proteins, induced apoptotic cell death, supporting the results obtained by PI staining (Fig. 4A, B).

Apoptosis induced by the expression of the E-protein was further confirmed using PI/annexin V-FITC staining (Fig. 4E, F). The FACS plots (Fig. 4E) show that the E-protein induced apoptotic, but not necrotic cell death.

Combining E-, N-, or 3b proteins expression led to levels of cell death induction comparable to those observed with E-protein alone (data not shown).

To verify the expression of these proteins, we used available antibodies against the viral proteins N and E. Only those targeting the N-protein found to be highly specific, as demonstrated in the immunoblot (Fig. S2A). Using PCR with specific primers, we confirmed the expression of both N- and E-proteins, but not 3b (Fig. S2B, C). Thus, the lack of effect of the 3b protein may be due to it not being expressed for unknown reasons.

**Fig. 5:**
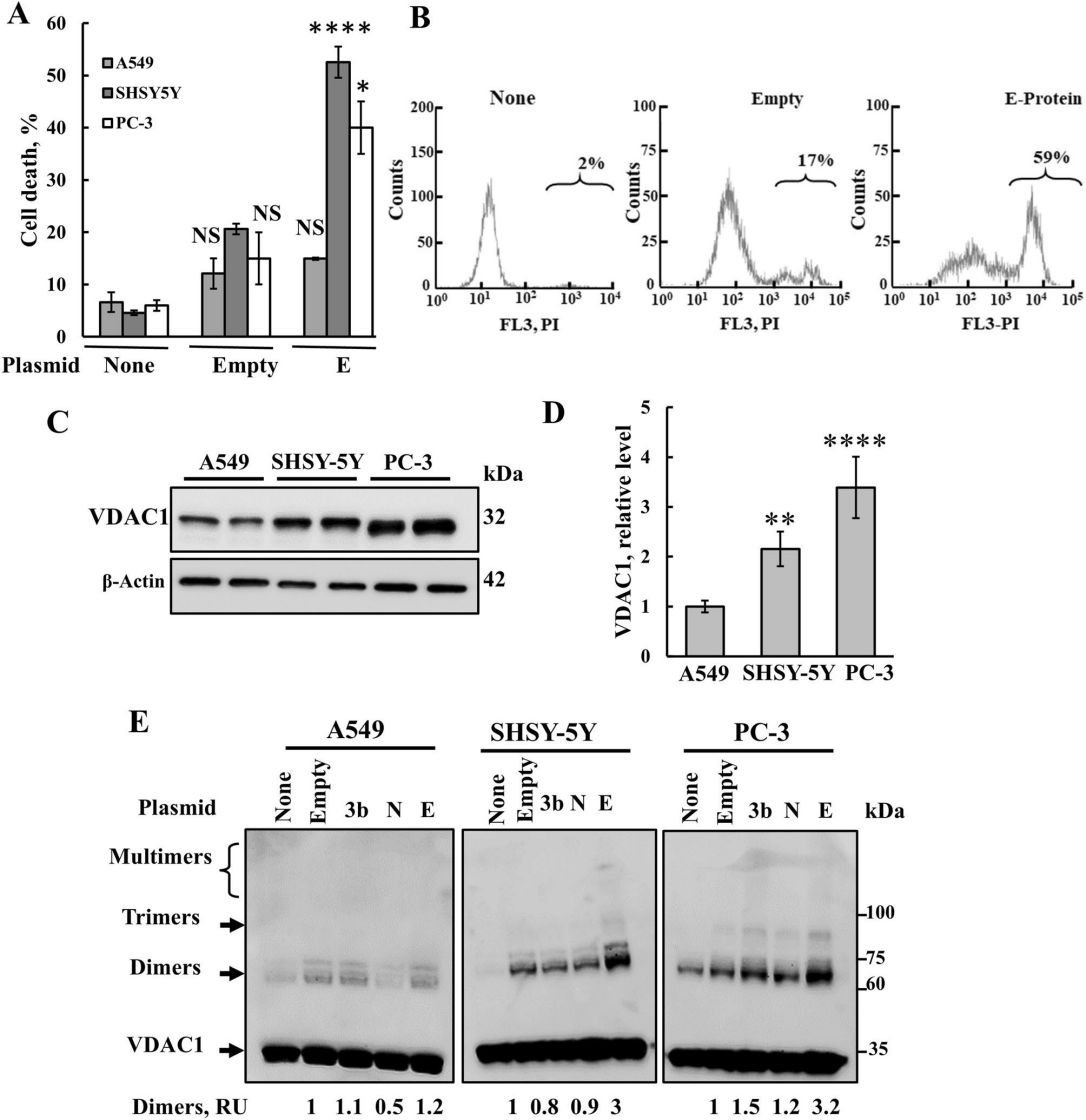
SARS-CoV-2 E-protein induces VDAC1 overexpression, and oligomerization is cell type-dependent. **A**, **B** A549, SHSY-5Y and PC-3 cells were transfected with 2 μg of pcDNA3.1 plasmid (empty) or encoding for proteins E, N, or 3b, and 48h post transfection, cells were analyzed for cell death using PI staining and flow cytometry (**A**). Representative FACS histograms are shown (**B**). **C**–**E** Transfected cell lines A549, SHSY-5Y, and PC-3 were analyzed for VDAC1 expression levels by immunoblotting (**C**) and quantification (**D**) and subjected to cross-linking using EGS (1mg protein/ml,100μM) (**E**). The positions of VDAC1 monomers, dimers, trimers, and multimers and of the molecular weight standards are indicated. VDAC1 dimer levels are presented relative to their levels in the empty plasmid-transfected cells (RUs) and are shown below the immunoblot. Results represent the means ± SEM (n = 3) *p ≤ 0.05; **p ≤ 0.01; ****p ≤ 0.0001; *NS* non-specific

The induction of cell death by the expressed E-protein exhibited cell type dependency as demonstrated using lung cancer A549, prostate cancer PC-3, and neuroblastoma SHSY-5Y cells. The highest levels of apoptosis were observed in SHSY-5Y and PC-3 cells, with lesser induction observed in A549 cells (Fig. 5A, B).

Analysis of E-protein-induced VDAC1 overexpression, oligomerization, and apoptosis revealed that SHSY-5Y and PC-3 cells exhibited the highest levels of VDAC1, whereas A549 cells showed lower levels (Fig. 5C–E). Similar results were obtained for E-protein-induced VDAC1 oligomerization (Fig. 5E). Thus, SHSY-5Y was selected for most of the performed studies.

The effects of E-protein expression on the three different cell lines (Fig. 5) support the association between VDAC between VDAC1 expression levels, oligomerization, and apoptosis.

### Expression of E-protein reduced ROS production and induced an increase in cytosolic Ca^2+^ and fmtDNA release

**Fig. 6:**
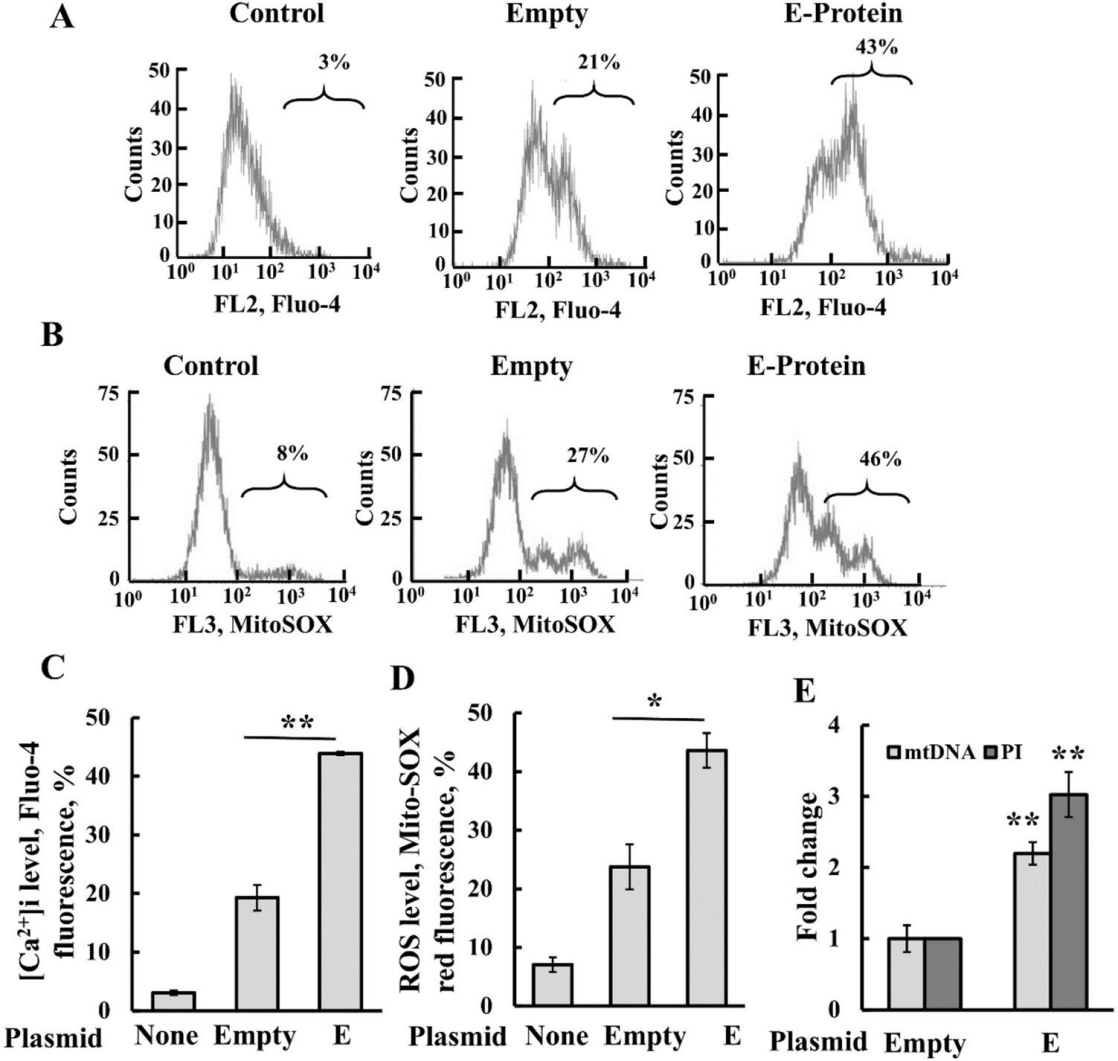
E-protein increases intracellular Ca^2+^ levels, ROS production and mtDNA release (**A**–**D**) SHSY-5Y cells were transfected with 2 μg of pcDNA3.1 plasmid (empty) or encoding for protein E, and 48h post transfection, cells were subjected to analysis of the levels of intracellular Ca^2+^ using Fluo-4 (**A**, **C**), mitochondrial ROS with MitoSOX-Red (**B**, **D**), followed by flow cytometry. Representative FACS histograms (**A**, **B**) and quantitative analysis (**C**, **D**) are shown. **E** Cytosolic fmtDNA levels were analyzed as indicated in the Methods section. Cells were transfected with 1.5 μg of pcDNA3.1 plasmid (empty) or encoding for E-protein, and 48h post-transfection, analyzed for fmtDNA using qPCR and primers specific to the D-Loop-II. Cells were also subjected to cell death analysis using PI staining and flow cytometry. Results represent the means ± SEM (n = 3) *p ≤ 0.05; **p ≤ 0.01

Since apoptosis is often accompanied by mitochondria dysfunction, we examined the impact of the expressed E-protein on cellular Ca^2+^ levels and mitochondrial superoxide using MitoSOX (Fig. 6A–D). Cell transfected to express E-protein exhibited elevated cytosolic Ca^2+^ ([Ca^2+^]i) levels, as monitored using Fluo-4-AM (Fig. 6A, C). Similarly, ROS levels were also increased (Fig. 6B, D).

Recently, we showed that fmtDNA is released from the mitochondria via oligomeric VDAC1, and that VBIT-4 inhibits fmtDNA release, type-Ι interferon signaling, and disease severity in a mouse model of lupus [23]. Given that the E-protein led to VDAC1 overexpression and oligomerization, we investigated whether it also triggers fmtDNA release into the cytosol (Fig. 6E). The results reveal that the E-protein does indeed induce this release.

### Interaction of the recombinant viral E- and N-proteins with purified VDAC1 and MAVS

**Fig. 7:**
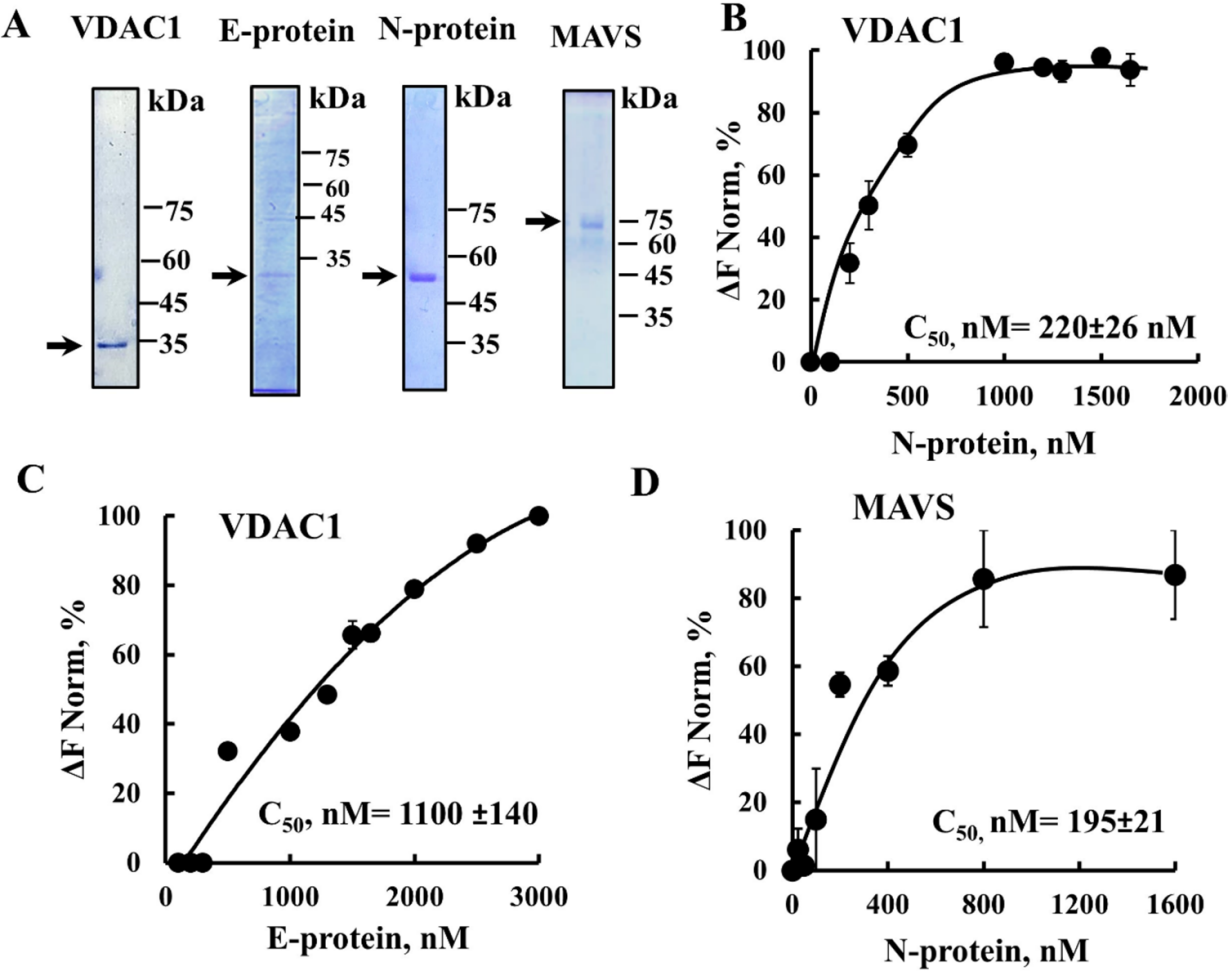
Interaction of SARS-CoV-2 purified N- and E-proteins with purified VDAC1 and MAVS. **A** SDS-PAGE of purified VDAC1, MAVS, and SARS-CoV-2 E- and N-proteins stained with Coomassie blue. **B**–**D** The interactions of VDAC1 with purified E- or N-protein (**B**, **C**) and of MAVS with the N-protein (**D**) were analyzed using MST, as described in the Methods section. The concentration of E- or N-protein giving 50% of the maximal binding (C50) is indicated

The direct interaction of purified VDAC1 and purified MAVS with the purified viral N- and E-proteins (Fig. 7A) was analyzed using Microscale thermophoresis (MST) [24] (Fig. 7B–D). Both proteins interacted with VDAC1 with a half maximal binding (C_50_) of 220 ± 26 nM and 1100 ± 140 nM for the N- and E-proteins, respectively. This suggests relatively high-affinity binding, with the N-protein displaying higher binding affinity than the E-protein (Fig. 7B, C).

The mitochondrial antiviral protein MAVS is anchored to the OMM and plays an important role in innate immunity. It also contributes to the formation of an antiviral and signaling immune system by activating pro-inflammatory cytokines to combat virus infection within the cell [59, 60].

Given that the MAVS–VDAC1 interaction may underline MAVS’s action as an antiviral protein [61] and its significance in innate immunity, we investigated MAVS binding to the N-protein. The findings demonstrate that MAVS indeed interacts with the N-protein, exhibiting high affinity with a C_50_ of 195 ± 21 nM (Fig. 7D).

## Discussion

SARS-CoV-2 invades the mitochondria of infected cells, resulting in altered mitochondrial signaling, modifies cellular energy metabolism, induces apoptosis, mitophagy, and abnormal levels of mitochondrial proteins [62]. The link between VDAC1 and SARS-CoV-2 was demonstrated, by the overexpression of VDAC1 in a population of T cells, suppression of mtDNA release, cGAS-STING pathway activation and improved survival in the T cells of COVID-19 patients when treated with the VDAC1-interacting compound VBIT-4 [33]. mtDNA plays a critical role in cGAS-STING activation during SARS-CoV-2 infection, and VBIT-4 has been shown to protect against this activation [25]. Moreover, elevated levels of circulating fmtDNA are associated with an increased probability of intensive care unit admission higher mortality risk and disease severity, influencing the immune response in patients [23, 44, 63–66].

Additionally, VDAC1 levels were found to be decreased in extracellular vesicle isolated from the blood of acutely infected patients but not in those with long COVID-19 [67].

### Elevated fmtDNA in serum of COVID-19-infected patients

Alterations in mtDNA content have been shown to disrupt cell metabolism and impair innate immune responses, contributing to inflammatory pathology, including the upregulation of interferon-stimulated genes [68]. Numerous reports have highlighted the association between the presence of mtDNA outside the mitochondria and various human disorders that include neuromuscular and neurodegenerative diseases, cancer, diabetes, cardiovascular disorders, and systemic lupus erythematosus [69].

Viruses can manipulate host-cell mitochondria and damage mtDNA to exert control over cellular functions. For example, the Zta protein encoded by the Ebola virus impacts mtDNA replication [70]. Hepatitis C virus (HCV) infection results in mtDNA damage, and the herpes simplex virus produces UL12.5 protein, which leads to mtDNA degradation [71]. Moreover, HIV and HCV infections cause mtDNA depletion in co-infected patients [72].

Previous studies have demonstrated that in COVID-19 patients elevated levels of circulating cell-free fmtDNA are associated with an increased probability of intensive care unit admission and heightened risk of death [64–66]. Furthermore, fmtDNA was found in the plasma of COVID-19 patients, with high mtDNA levels correlated with disease severity, and influencing the immune response in these patients [23, 44, 63]. These findings underscore the potential use of fmtDNA levels as a prognostic marker for COVID-19 severity and outcomes.

Here, we analyzed serum fmtDNA levels in both severe and non-severe COVID-19 patients and found a significant increase in fmtDNA levels in their serum compared to healthy donors (Fig. 1). Specifically, the levels of the fmtDNA displacement loop (D-loop)-II, a non-coding region, showed a 24-fold increase, while COX-III and ND-2 exhibited increases of 4- and sevenfold, respectively.

However, we did not observe a clear relationship between COVID-19 serum fmtDNA levels and disease severity. Critically ill COVID-19 patients displayed an early increase in plasma levels of ND1 fmtDNA, with a peak at 24h [44]. This may explain why in our study no clear correlation was found between disease severity and fmtDNA levels, as the blood samples were taken from patients at different time points following disease onset.

**Fig. 8:**
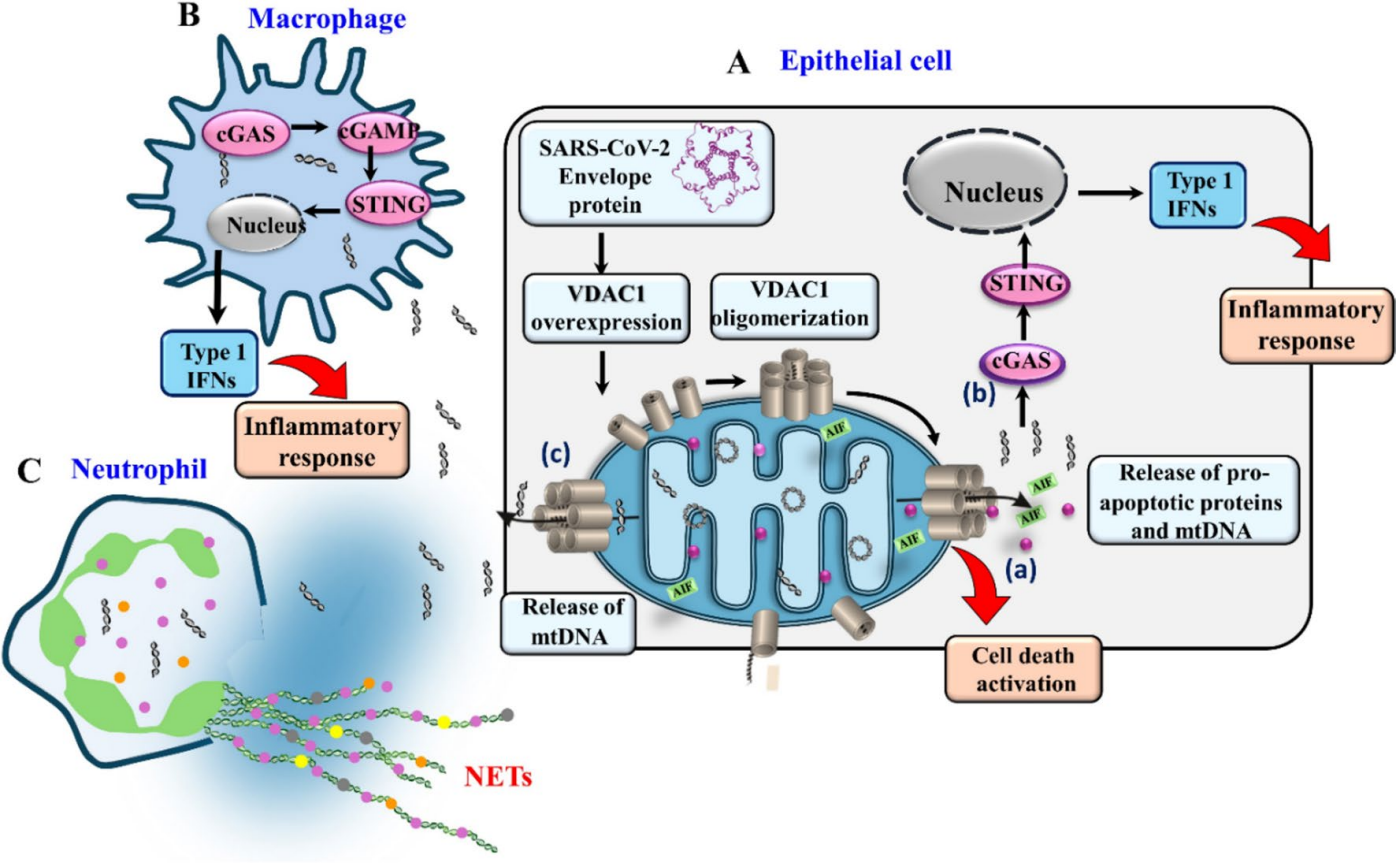
Proposed model for SARS-CoV-2 E-protein inducing VDAC1 overexpression and oligomerization, cell death, and inflammation activation. **A** Epithelial cell expressing the SARS-CoV-2 E-protein results in VDAC1 overexpression and VDAC1 oligomerization, forming a large channel mediating the release of apoptogenic proteins such as cytochrome c (Cyto c) and apoptosis-inducing factor (AIF), leading to cell death and of fmtDNA (**a**). Released fmtDNA activates the cyclic GMP-AMP synthase (cGAS)-STING pathway, leading to induction of expression of a type 1 interferon and inflammatory response (**b**). The pathways in **a** and **b** during SARS-CoV-2 infection play a role in epithelial cell death and pulmonary inflammation, with fmtDNA acting to induce an inflammatory response [25]. **B**. When macrophages engulf the dead cell debris, including the mtDNA released to the cytosol (**c**), cGAS-STING signaling is triggered in these cells, causing the macrophages to produce a large amount of IFN that results in a robust inflammatory response. **C** Extracellular mtDNA can also activate neutrophil extracellular traps (NETs). Thus, SARS-CoV-2 infection causes mitochondrial damage in epithelial cells, resulting in the release of fmtDNA to the cytosol, which activates the cGAS-STING pathway and causes excessive inflammation, affecting lung pathology in the late stage of SARS-CoV-2 infection. mitochondria/VDAC1

The mechanism underlying fmtDNA release in response to SARS-CoV-2 remains unclear. Based on recent findings demonstrating that fmtDNA is released from the mitochondria via oligomeric VDAC1 [23], we suggest that a similar mechanism would be applied in COVID-19 (Fig. 8). We showed that the VDAC1 oligomerization inhibitor, VBIT-4, effectively inhibits fmtDNA release, type-I interferon signaling, and disease severity in a mouse model of lupus [23]. Similarly, VBIT-4 promotes survival in the T cells of COVID-19 patients [33]. The roles of VDAC1 in fmtDNA release, promoting cell death and inflammation and involvement in the pathogenesis of COVID-19 are discussed below.

### Different serum protein profile of COVID-19-infected patients compared to healthy people

A number of biomarkers have been identified for COVID-19 [73, 74]. These include several proinflammatory cytokines, neuron-specific enolase, lactate dehydrogenase (LDH), aspartate transaminase (AST), neutrophil count, troponins, creatine kinase, myoglobin, D-dimer, and brain natriuretic peptide, among others [75]. Monitoring these molecular markers can provide valuable insights into diagnosis, prognosis, and severity, and outcomes of the disease in COVID-19 patients, aiding in clinical management and decision making.

A recent proteomic analysis of extracellular vesicles found in the saliva of COVID-19 patients uncovered a protein signature associated with immune response processes, oxygen transport, and antioxidant mechanisms [76].

Our SDS-PAGE analysis of sera from COVID-19 patients and healthy donors revealed three major protein bands with molecular masses of 49, 17, and 14 kDa, defined as unique to patients, where their serum includes either two or all three of the proteins (Fig. 2, Table 2). Subsequent proteomic analysis using LC–MS/MS, has identified over 20 proteins within these bands, each distinguished by several unique peptides. From these, we selected 15 proteins with the highest sequence coverage and scores, all displaying very high levels compared to those in healthy patients (Table 2).

Among these proteins were: haptoglobin, that forms a complex haptoglobin–hemoglobin that is subsequently removed by the reticuloendothelial system [45]. It is associated with inflammation and numerous diseases [77]; CD5 antigen-like (CD5L), secreted primarily from macrophages during an inflammatory response [48]. It is associated with autophagy regulation, cell polarization, apoptosis inhibition, and lipid metabolism regulation, and is implicated in various diseases resulting from acute or chronic inflammation, including infectious, metabolic, and autoimmune conditions [78]; apolipoprotein A-1 (ApoA1), a major protein constituent of high-density lipoprotein (HDL) particles linked to the pathogenesis of cardiometabolic diseases, atherosclerosis, thrombosis, diabetes, cancer, and neurological disorders [79]; transthyretin (TTR), which transports thyroxine and retinol-binding protein (RBP) bound to retinol [55]. Its misfolding and aggregation can lead to amyloid transthyretin (ATTR) formation. Patients with ATTR amyloidosis are particularly vulnerable to COVID-19 [80]; retinoic acid receptor responder 2 (CRABP-II), involved in transporting retinoic acid (RA) from the cytosol to the nucleus, enhancing cell transcriptional activity [53]; retinol-binding protein 4 (RBP4), which transports retinol from liver stores to peripheral tissues [54]. When associated with TTR, the retinol/RBP4/TTR complex is released into the bloodstream to deliver retinol to tissues [54]. We found an increase in both RBP4 and TTR in the serum of COVID-19 patients (Table 2), consistent with the secretion of this complex into circulation [54]; apolipoprotein C-IV (APOC4), a lipid-binding protein critical for activating lipoprotein lipase [57]; and immunoglobulin kappa constant (IGKC), an immunoglobulin isotype involved in immune response pathways such as the Fc epsilon RI pathway and NFAT in immune response.

Other detected proteins have previously been reported as elevated in COVID-19 patients’ serum. Among these is C-reactive protein (CRP), with an increase of over 290-fold (Table 2). This is consistent with previous findings of elevated CRP levels in the serum of COVID-19 patients [81, 82] being correlated with COVID-19 severity [83], suggests it as a potential early marker for predicting disease severity [84, 85]. CRP functions by activating the classic complement pathway through binding to phosphocholine expressed on bacterial cell surfaces, such as pneumococcus bacteria [86].

Another protein with markedly increased levels (274-fold) in COVID-19 patients is the complement component C8-beta chain, a crucial constituent of the membrane attack complex (MAC) responsible for mediating cell lysis and affecting cell membrane integrity [46], playing a role in both innate and adaptive immune responses.

Levels of alpha-1-antichymotrypsin (AACT**)** protein were found to be increased 23-fold. AACT functions as a serine protease inhibitor and acts in bacterial killing by neutrophils and is induced during inflammation [87].

Similarly, levels of the serine protease inhibitor alpha-1-anti-trypsin (AAT), which protects the lungs from neutrophil elastase damage [51] were increased by 26-fold, possibly reflecting the body’s response to protect the lungs. Levels of immunoglobulin Joining chain (J chain) showed an increase of 21-fold. This protein is essential for the formation and stabilization of polymeric Ig structure [52], indicating its potential role in the immune response in COVID-19 patients.

These findings indicate the presence in the serum of COVID-19 patients of proteins associated with both innate and adaptive immune responses, and inflammatory processes. This aligns with the pathophysiological mechanisms of COVID-19, which heavily involve the immune system. [88].

### COVID-19 proteins induced apoptosis, inflammation, and mitochondria dysfunction via induction of VDAC1 overexpression and its oligomerization

Numerous viruses exert their pathogenic effects by disrupting mitochondrial activities, either directly targeted by viral proteins or influenced by alterations in their cellular milieu, deregulated Ca^2+^ homeostasis, ER stress, and/or apoptosis [89]. The connection between mitochondrial dysfunction and SARS-CoV-2 infection, including hijacking of host mitochondria [90], altering mitochondrial metabolism [91], and inducing mtDNA release [25] has been demonstrated.

Here, as mitochondria dysfunction by viruses often involves modulation of VDAC1 expression levels or its activity via the interaction of their specific proteins with VDAC1 [26–30, 92, 93], we aimed to elucidate the role of VDAC1 in COVID-19 pathology. We selected four SARS-CoV-2-encoded proteins and proposed to mediate their pathology via inducing mitochondria dysfunction and apoptosis. We illustrated their effects on VDAC1 expression levels, promoting its oligomerization, and inducing apoptosis.

Of the three SARS-CoV-2 proteins E, 3b, and N expressed in cells, the E-protein induced VDAC1 overexpression and its oligomerization, increased ROS production, and elevated intracellular Ca^2+^ levels, apoptosis, and the release of fmtDNA (Figs. 3–6). The E-protein, known for altering the permeability of host-cell membranes and being implicated in the inflammatory cascade triggered by SARS-CoV [34], exhibited the most significant activity.

The proposed pathways activated by SARS-CoV-2 E-protein leading to activation of apoptosis and an inflammatory response are presented in Fig. 8. E-protein induces VDAC1 overexpression and its oligomerization, resulting in the formation of a large channel that mediates the release of pro-apoptotic proteins [16, 18, 20, 22, 24] and mtDNA [23] from the mitochondria to the cytosol, leading to apoptosis activation and an inflammatory response (Fig. 8A).

Lymphocytes from COVID-A patients exhibit mitochondrial dysfunction, a distinct metabolic profile, and are susceptible to apoptosis due to elevated VDAC1 levels [33]. Additionally, a specific T-cell subset observed in COVID-A patients displays overexpression of both VDAC1 and H3K27me3, an epigenetic marker. The survival of these cells was rescued by VBIT-4, an inhibitor of VDAC1 oligomerization [33]. These findings align with VBIT-4’s ability to suppress VDAC1 oligomerization resulting from its overexpression, thereby preventing cell death [19]. Since VDAC1 oligomers mediate mtDNA released from the mitochondria [23], as expected, the E-protein stimulated fmtDNA release (Fig. 6E).

VDAC1 overexpression, its oligomerization, and apoptosis were triggered by various apoptosis-inducing conditions including chemotherapy drugs and UV irradiation [16–18, 20, 94] in several disorders such as Alzheimer’s disease [95], type 2 diabetes [96], and autoimmune diseases such as lupus [23] and inflammatory bowel diseases [97]. Also, several viral proteins elevate VDAC1 expression [28, 33, 92]. Hence, similar to chemotherapy drugs, stress conditions, various diseases, and viruses, SARS-CoV-2 E-protein induces VDAC1 overexpression.

The increase in VDAC1 levels upon E-protein expression may result from increased intracellular Ca^2+^ levels (Fig. 6C), which are required for VDAC1 overexpression induced by various chemicals and conditions [20, 98]. Additionally, since purified protein E directly interacts with VDAC1 (Fig. 7C), it may stabilize VDAC1 and prevent its degradation.

### Direct interaction of purified E-and N-proteins with VDAC1 and MAVS

Here, we showed that both the purified E- and N-proteins interact directly with purified VDAC1 (Fig. 7). However, the precise function of these interactions remains unclear. It is possible that the E-protein may directly stimulate VDAC1 oligomerization to form a larger channel, as we found with mtDNA [23] and amyloid beta (Aβ) [99]. On the other hand, the interaction of the N-protein with VDAC1 may modulate VDAC1 activity, potentially involving functions unrelated to cell death, but associated with other multifunctional roles of VDAC1.

We also showed interaction of the N-protein with the mitochondrial protein MAVS, which plays a crucial role in antiviral responses, and signals the immune system by activating pro-inflammatory cytokines to combat viral infections within cells [59]. MAVS exerts its activity only when associated with the mitochondria [60]. Numerous viruses were shown to displace MAVS from the mitochondria, thereby impeding the IFN-I response [100, 101] and escape from host defenses [60]. Recently [61], we demonstrated that MAVS interacts with VDAC1 and suggested that this interaction lies at the base of MAVS’ antiviral action. The N-protein, by binding to VDAC1 or/and MAVS potentially hinders MAVS’ signaling to the immune system through MAVS’ detachment from the mitochondria/VDAC1.

### Cytosolic and extracellular mtDNA in patients with COVID-19 and dysregulation of the immune and inflammatory systems

In this study, we demonstrated elevated levels of fmtDNA in the serum of COVID-19 patients and in the cytosol of cells expressing E-protein. The release of mtDNA reflects the formation of a large channel associated with VDAC1 oligomerization that mediates the mtDNA release [23] and of pro-apoptotic proteins [16, 18, 20, 22, 24], resulting in cell death and inflammation (Fig. 8).

We propose that VDAC1 overexpression and the formation of a mega channel through its oligomerization lead to fmtDNA release and activate the cGAS–STING pathway that triggers type I interferon responses (Fig. 8A). This is supported by findings that the VDAC1 oligomerization inhibitor, VBIT-4, protected against cGAS-STING activation in the cytokine storm derived from SARS-CoV-2 infection [25].

When mtDNA is released from damaged or dying cells into the cytoplasm or circulation, it can impact immunity [102], as shown by mtDNA’s influence on the immune response in COVID-19 [44, 63–66]. Extracellular mtDNA can activate macrophages when they engulf the dead cell debris into the cytosol. This triggers cGAS-STING signaling in these cells, causing the macrophages to produce a large amount of IFN, resulting in an inflammatory response (Fig. 8B). Extracellular fmtDNA can also activate neutrophils leading to the release of web-like structures—neutrophil extracellular traps (NETs) (Fig. 8C). High levels of NETs were found in both the peripheral blood and lung tissues of COVID-19 patients and contribute to disease pathogenesis [103–106]. Activated neutrophils also release lytic enzymes, ROS, cytokines, histones, mtDNA, and nucleus (nDNA) [107]. mtDNA within NETs contribute to the function of these extracellular traps in host defense mechanisms [108] (Fig. 8C).

Consequently, the release of mtDNA into the cytoplasm and extracellular environment triggers various pattern recognition receptors that contribute to the immune response, promoting an inflammatory cascade in patients with COVID-19 [109].

While this study did not directly show that SARS-CoV-2 infection or the E-protein induces mtDNA release and activates the cGAS-STING pathway and a cytokine storm, the connection between mtDNA release and cGAS-STING activation is well established [110–112]. Additionally, it has been demonstrated that VBIT-4, which inhibits mtDNA release and cGAS-STING pathway activation, as well as interferon-stimulating genes [23], protects against cGAS-STING activation in SARS-CoV-2 infection [25].

In summary, our study revealed elevated levels of mtDNA in the serum of COVID-19 patients. Additionally, we demonstrated that the E-protein of COVID-19 enhances the overexpression and oligomerization of VDAC1, which leads to cell death. Furthermore, VDAC1 oligomers mediate the release of mtDNA into the cytosol, where it triggers the activation of toll-like receptors and the cGAS-STING signaling axis. This activation prompts the expression of various inflammatory cytokines and chemokines in a cytokine storm, which is associated with severe tissue damage in infectious or non-infectious conditions, particularly in viral respiratory infections such as H5N1 influenza, SARS-CoV-1, and SARS-CoV-2 [113, 114]*.*

The findings presented here suggest that COVID-19 pathology is associated with the induction of VDAC1 overexpression and oligomerization, leading to cell death and the release of fmtDNA, which is associated with the cytokine storm that directly influences SARS-CoV-2 severity and the COVID-19 illness.

## Supplementary Information

Below is the link to the electronic supplementary material.

Supplementary file1 (PDF 491 KB)

## Data Availability

No datasets were generated or analysed during the current study.
