# Correction to: Elevated serum mtDNA in COVID‑19 patients is linked to SARS‑CoV‑2 envelope protein targeting mitochondrial VDAC1, inducing apoptosis and mtDNA release

**DOI:** 10.1007/s10495-025-02113-0

**Published:** 2025-05-10

**Authors:** Anna Shteinfer‑Kuzmine, Ankit Verma, Rut Bornshten, Eli Ben Chetrit, Ami Ben‑Ya’acov, Hadas Pahima, Ethan Rubin, Yosef Mograbi, Eyal Shteyer, Varda Shoshan‑Barmatz

**Affiliations:** 1 National Institute for Biotechnology in the Negev, Beer‑Sheva, Israel; 2 Department of Life Sciences, Ben-Gurion University of the Negev, Beer‑Sheva, 84105 Israel; 3 The Shraga Segal Dept. of Microbiology, Immunology and Genetics, Ben-Gurion University of the Negev, Beer‑Sheva, 84105 Israel; 4 Infectious Diseases Unit, Shaare Zedek Medical Center, Hebrew University School of Medicine, Jerusalem, Israel; 5 Shaare Zedek Medical Center, The Juliet Keidan Institute of Paediatric Gastroenterology, Jerusalem, Israel; 6 Tel Aviv‑Yafo, Israel

**Correction to: Elevated serum mtDNA in COVID‑19 patients is linked to SARS‑CoV‑2 envelope protein targeting mitochondrial VDAC1, inducing apoptosis and mtDNA release, Apoptosis (2024) 29:2025–2046**. 10.1007/s10495-024-02025-5.

In this article, Anna Shteinfer Kuzmine and Ankit Verma should have been designated as co-first authors, as both contributed equally to this work.

The original article has been corrected.

