# Supplementary material for: Elevated serum mtDNA in COVID-19 patients is linked to SARS-CoV-2 envelope protein targeting mitochondrial VDAC1, inducing apoptosis and mtDNA release
Source: Apoptosis. 2024 Oct 7;29(11-12):2025–46. doi: 10.1007/s10495-024-02025-5 (PMC11550248; doi:10.1007/s10495-024-02025-5)
Supplement: Supplementary file 1 — Supplementary file1 (PDF 491 KB) [file 10495_2024_2025_MOESM1_ESM.pdf]

Supplementary Materials

Table S1. Clinical sample information

Demographic, clinical, and selected lab test characteristics of COVID-19 patients. CRP=C-reactive protein, HFNC=high flow nasal canula, RA=room air, FM=face mask, MV=mechanical ventilation, NC=nasal cannula

No.	Gender	Age	Onset of symptoms	%Sat	O2 support	Severity of disease	CRP (mg/dL)	Ferritin (ng/mL)	Absolute Lymph count (10^3/uL)	D-dimer (ng/mL)
**P1**	Male	56	<10 days	85	RA	Severe	19.00	NA	1.10	
**P2**	Male	62	>10 days	91	RA	Moderate	0.34	NA	1.00	628
**P3**	Male	65	<10 days	95	RA	Mild	8.90		0.66	
**P4**	Male	57	<10 days	94	RA	Moderate	2.80		0.73	
**P5**	Male	66	<10 days	< 90	HFNC	Severe	10.70		0.96	622
**P6**	Male	60	<10 days	< 90	HFNC	Severe	2.30		1.08	1790
**P7**	Male	41	<10 days	< 90	HFNC	Severe	14.20	2120	0.78	1509
**P8**	Female	58	<10 days	96	RA	Mild	0.3	NA	2.6	
**P9**	Female	64	>10 days	94	RA	Moderate	3.9	NA	1.4	286
**P10**	Male	52	<10 days	90	NC	Severe	24.8	1332	1.25	
**P11**	Male	47	<10 days	94	RA	Mild			1.70	
**P12**	Female	43	<10 days	97	RA	Mild	6.00		1.50	483
**P13**	Male	74	<10 days	< 90	HFNC	Severe	23.60	830	0.65	1733
**P14**	Male	48	<10 days	94	RA	Mild	7		1.05	784
**P15**	Male	48	<10 days	< 90	HFNC	Severe	20	465	0.77	998
**P16**	Male	57	<10 days	93	RA	Moderate	NA		1.6	
**P17**	Male	58	<10 days	< 90	HFNC	Severe	4.8		2.1	413
**P18**	Female	74	<10 days	93	RA	Moderate	2.7		1.48	1258
**P19**	Male	23	<10 days	98	RA	Moderate	NA			
**P20**	Male	69	<10 days	< 90	MV	Critical	24.00	136	1.38	11595
**P21**	Female	99	<10 days	95	100% FM	Critical	19.00		1.00	
**P22**	Female	61	<10 days	88	RA	Severe	10.90	1685	0.55	451
**P24**	Male	60	<10 days	96	RA	Mild	3.70	586	0.48	586
**P25**	Female	40	<10 days	96	RA	Mild	5.7	70	1.06	154
**P26**	Female	55	<10 days	91	NC	Severe	14.78	244	1.78	
**P27**	Female	23	<10 days	96	RA	Mild			1.7	
**P28**	Female	31	>10 days	96	RA	Mild			1.30	
**P29**	Male	77	>10 days	< 90	MV	Severe	15.00		0.55	4995
**P30**	Male	48	<10 days	94	RA	Mild	9.40		0.95	
**P31**	Female	89	<10 days	83	RA	Severe	2.98			
**P32**	Female	77	<10 days	94	RA	Mild	9.70		0.67	
**P33**	Female	58	<10 days	96	NC	Severe	10.80	137	1.94	298
**P34**	Male	58	<10 days	93	NC	Severe	24.60	768	0.60	1166
**P35**	Male	64	<10 days	93	RA	Mild	6.00	469	1.50	10035
**P36**	Female	30	<10 days	94	RA	Mild	0.16		1.04	193
**P37**	Female	80	<10 days	96	RA	Moderate	7.10	306	0.70	580
**P38**	Female	59	<10 days	97	RA	Mild	0.20		1.10	626

**P39**	Male	51	<10 days	96	RA	Mild	1.60		1.67	183
**P40**	Female	76	<10 days	94	RA	Mild	3.2	101	1.48	
**P41**	Female	87	<10 days	94	NC	Moderate	1.73	295	0.5	
**P42**	Female	93	<10 days	92	RA	Severe	3.75		2.9	2333
**P43**	Male	87	<10 days	90	RA	Severe	18.7		0.7	
**P44**	Male	57	<10 days	94	RA	Moderate	9		0.7	
**P45**	Male	76	<10 days	90	RA	Severe	4.20		1.30	
**P46**	Female	90	<10 days	93	NC	Severe	18.70		1.90	313
**P47**	Female	84	<10 days	90	100% FM	Critical	14.30	670	0.60	
**P48**	Male	87	<10 days	< 90	HFNC	Severe	32.00	821	0.59	1384
**P49**	Female	76	<10 days	93	NC	Severe	0.50	85	2.80	
**P50**	Male	62	<10 days	98	RA	Mild	2.90	528	1.04	299
**P51**	Male	71	<10 days	93	RA	Moderate	17.00		1.20	2016
**P53**	Female	67	<10 days	90	NC	Severe	4.00	143	0.75	

Table S2. Gene specific q-PCR primers used in the study

Gene name	Primers
Loop-II	Forward TCAACTGCAACTCCAAAGCC<br>Reverse GGGGACGAGAAGGGATTTGA
Loop -I	Forward CGTACATTACTGCCAGCCAC<br>Reverse GGCTTTGGAGTTGCAGTTGA
COX-III	Forward ATGACCCACCAATCACATGC<br>Reverse ATCACATGGCTAGGCCGGAG
ND-2	Forward CACAGAAGCTGCCATCAAGTA<br>Reverse CCGGAGAGTATATTGTTGAAGAG
E	Forward TCGTTTCGGAAGAGACAGGT<br>Reverse ACTAGCCATCCTTACTGCG
N	Forward AGAATGGAGAACGCAGTGGGG<br>Reverse GTCTTGGTTCACCGCTCTCA
3b	Forward TGATGCCAACTATTTTCTTTG<br>Reverse TATTGTAAGGTATACAATAG
β-Actin	Forward CATTGCTGACAGGATGCAGAAGG<br>Reverse TGCTGGAAGGTGGACAGTGAGG

Table S3. Result of a Machine Learning model to predict disease outcomes from mtDNA alone. A predictive model was built with a leave-one-out approach (n=34). The confusion matrix compared predicted and actual outcomes.

		**Predicted**			
		**Healthy**	**Mild**	**Moderate**	**Severe**
**Actual**	Healthy	24	0	0	0
	Mild	0	23	1	5
	Moderate	0	0	7	0
	Severe	0	5	2	4

Description of proteomic-identified proteins found at high levels in COVID-19 patients: their function and relation to diseases

The p49 protein band comprises a number of proteins. These include:

Haptoglobin—a glycoprotein primarily secreted by the liver that binds to hemoglobin, where the haptoglobin–hemoglobin is removed by the reticuloendothelial system [1]. This protein plays a critical role in tissue repair, and regeneration and prevention of oxidative damage, and it is proposed as a biomarker of various pathologies [1]. Haptoglobin is linked to inflammation and many diseases [2], such as diabetic nephropathy [3], Crohn's disease [4], Parkinson' disease [5], and others.

Complement component C8-beta chain—one of three subunits C8α (M_r ~ 64,000), C8β (M_r ~ 63,500), and C8γ (M_r ~ 20,400) of the complement component 8 (C8) protein. C8 is one component of the membrane attack complex (MAC), which mediates cell lysis and initiates membrane penetration of the complex. C8a and C8b are involved in the formation of the MAC and affect cell membrane integrity [6]. While C8g belongs to the lipocalin family, it is not essential for the MAC-mediated cytolytic activity that kills gram–negative bacteria [7]. The MAC plays a key role in the innate and adaptive immune response by forming pores in the plasma membrane of target cells. A rare defect of the complement classical pathway is associated with susceptibility to severe recurrent infections.

Alpha-1-antichymotrypsin (AACT, SERPINA3)—a serine protease inhibitor that is mainly synthesized in the liver, secreted into the blood, and then induced during inflammation. Its main physiological target is neutrophil cathepsin G, an enzyme that plays a key role in killing of bacteria by the neutrophils. AACT may serve as a diagnostic or prognostic biomarker or therapeutic target in treating tumors [8].

Serum albumin—a 65kDa globular, water-soluble protein that is synthesized by liver hepatocytes and is rapidly excreted into the bloodstream. Albumin is the most abundant protein found in mammal blood and has a very diverse role. It is essential for maintaining plasma oncotic pressure, and it functions as a plasma carrier protein for steroids, hemin, fatty acids, and thyroid hormones. As an anionic protein, albumin binds calcium in blood serum. Its low levels (hypoalbuminia) can result from a serious underlying medical condition, such as kidney, liver, or heart failure [9].

<u>The p17 protein band comprises the following proteins:</u>

C-reactive protein (CRP)—a pentameric protein synthesized by the liver. CRP has both proinflammatory and anti-inflammatory properties. It is the prototypical acute phase serum protein, which can rise rapidly in response to inflammation and infection [10], Markedly elevated levels of CRP are most often associated with infections [11] such as severe dengue infection [12]. CRP can activate the classic complement pathway by binding to phosphocholine expressed on the surface of bacterial cells such as pneumococcus. Thus, it is considered to be a relatively sensitive biomarker in infectious and non-infectious inflammation [13]. CRP has been found to trigger cell death in ischemic cells [14]. People infected with COVID-19 in Wuhan, China, had elevated levels of this protein [15, 16], *a*nd a correlation between serum CRP and COVID-19 severity has been proposed [17].

Apolipoprotein A-1 (ApoA1)—a main protein constituent of high-density lipoprotein (HDL) particles that has several well-documented cardioprotective functions. ApoA1 binding to lipids is induced by interaction between a highly hydrophobic C-terminal domain with the surface lipids of a particle. As ApoA1 is directly involved in lipid metabolism, this property is important for considering this apolipoprotein as one of the key molecular players in the pathogenesis of cardiometabolic diseases such as atherosclerosis. Beneficial functions of APOA1 in atherosclerosis, thrombosis, diabetes, cancer, and neurological disorders is increasing exponentially [18].

Alpha-1-anti-trypsin (AAT)—a serine protease inhibitor produced in the liver. Its primary function is to protect the lungs from neutrophil elastase which damages delicate lung tissue [19]. AAT deficiency is the most common hereditary disorder in adults [20]. It is associated with an increased risk of developing pulmonary emphysema and liver disease, and also skin problems. The increase observed may reflect the body's response to protect the lungs.

Immunoglobulin Joining chain (J chain)—a 137-residue polypeptide that essential in the formation and stabilization of polymeric Ig structures. It regulates multimerization of secretory IgM and IgA [21].

The p14 protein band includes:

Transthyretin (TTR or TBPA)—a 15.9 kDa that forms a tetrameric protein that transports the thyroid hormone thyroxine and the retinol-binding protein bound to retinol (vitamin A) [22]; TTR is mainly synthetized by the liver (13) and the choroid plexus of brain (14), which are the sources of this protein in plasma and cerebrospinal fluid (CSF), respectively. In humans, 90% of plasma TTR is secreted from liver after binding to the RBP-retinol complex before secretion into the plasma. The protein may also be involved in other intracellular processes including proteolysis, nerve regeneration, autophagy, and glucose homeostasis. TTR misfolding and aggregation forming amyloid transthyretin (ATTR) is known to be associated with amyloid deposition that predominantly affects the peripheral nerves or heart. Patients with ATTR amyloidosis represent a population particularly vulnerable to COVID-19 [23]. TTR is associated with several diseases [24], and functions in the preservation and regulation of memory function and behavior, as well as in protection against several types of neurodegeneration in AD models [25].

Serum amyloid A4 (SAA4)—a constitutive apolipoprotein of high-density lipoprotein. Different isoforms of SAA are expressed constitutively at different levels or in response to inflammatory stimuli. These proteins are produced predominantly by the liver. SAAs composed of two sub-groups, comprising acute-phase SAA1 and SAA2 are associated with HDL and inflammation [26], and SAA4 comprises >90% of the total serum SAA proteins [27]. SAA is also an acute phase marker that responds rapidly, increasing within hours after an inflammatory stimulus, and the magnitude of increase may be greater than that of C-reactive protein (CRP).

Apolipoprotein C-IV—also known as apolipoprotein C4 (APOC4), a lipid-binding protein belonging to the apolipoprotein gene family. It has a role in lipoprotein metabolism and is a critical cofactor for the activation of lipoprotein lipase (LPL), a plasma enzyme that hydrolyzes triglycerides (TGs) on TG-rich lipoproteins [28]. It is associated with diseases such as hyperlipoproteinemia and cardiovascular disease (CVD) [29].

Immunoglobulin kappa constant (IGKC)—immunoglobulin (Ig) isotypes are divided into classes and subclasses depending on the heavy chain they possess; IgM (μ); IgD (δ); IgG (γ) which encompasses subclasses IgG1, IgG2, IgG3, and IgG4; IgA (α) which encompasses subclasses IgA1 and IgA2; and IgE (ε) [30]. Each isotype is paired with either a kappa (κ) or lambda (λ) light chain to create a tetrameric immunoglobulin complex capable of triggering unique effector functions. Diseases associated with IGKC include immunoglobulin kappa light chain deficiency and amyloidosis. Among its related pathways are the immune response Fc epsilon RI pathway and immune response nuclear factor of activated T cells (NFAT).

CD5 antigen-like (CD5L)—also called apoptosis inhibitor of macrophage, is secreted primarily from macrophages in lymphoid tissues during an inflammatory response, and it regulates mechanisms in inflammatory responses, such as infection or atherosclerosis [31]. It is expressed in the spleen, lymph nodes, thymus, and bone marrow [32]. This protein acts as an inhibitor of apoptosis and as a key regulator of lipid synthesis, inducing lipolysis in the progression of obesity, and it participates in obesity-associated autoimmunity. Due to its multifaceted roles that range from pattern recognition to autophagy, cell polarization, and the regulation of lipid metabolism, CD5L plays a key role in highly prevalent diseases that develop due to either acute or chronic inflammation, including several infectious, metabolic, and autoimmune conditions [33].

Retinol-binding protein 4 (RBP4)—a transporter protein for retinol from the liver that is stored in the peripheral tissues [34]. In plasma, the RBP-retinol complex interacts with transthyretin that transport retinol from the plasma and cerebrospinal fluid to the liver. After associating with transthyretin (TTR), the retinol/RBP4/TTR complex is released into the bloodstream and delivers retinol to the tissues via binding to specific membrane receptor tissues [34]. We found that both **RBP4** and TTR were

increased in Covid-19 patients (Table 2). This was expected, as after the formation of the complex, retinol/RBP4/TTR is secreted into the circulation [34]. However, it is also reported that RBP4 levels were decreased in hospitalized patients with critical illness compared to nonpatients [35].

Cytosolic retinoic acid receptor responder 2 (CRABP-II)—a soluble protein and member of the family of intracellular lipid-binding proteins transporting retinoic acid (RA) from the cytosol to the nucleus where it directly associates with the RA receptor, thereby enhancing its transcriptional activity [36]. Its functions include antimicrobial activity against bacteria and fungi that [37]. Viral RNA recognition mechanism through RIG-I receptors can quickly consume a large amount of the body's retinoid reserve, which causes the retinol levels to fall below the normal serum levels. This causes retinoid insufficiency and impaired retinoid signaling, which leads to an interruption in Type I interferon synthesis and excessive inflammation. Interestingly, three of the identified proteins **CRABP-II, RBP4,** and **transthyretin** are related to retinoid signaling, and thereby the regulation of Type I interferon synthesis and excessive inflammation.

Fig. S1. The sequnces with their LC-MS/MS identified peptides of the proteins identified in the protein bands p49, p17 and p14

Protein band-p49

1. Haptoglobin – P00738

MSALGAVIALLLWGQLFAVDSGNDVTDIADDGCPKPPEIAHGYVEHSVRYQCKNYYKLRTEGDGVY
TLNDKKQWINKAVGDKLPECEADDGCPKPPEIAHGYVEHSVRYQCKNYYKLRTEGDGVYTLNNEK
QWINKAVGDKLPECEAVCGKPKNPANPVQRILGGHLDAKGSFPWQAKMVSHHNLTTGATLINEQW
LLTTAKNLFLNHSENATAKDIAPTLTLYVGKKQLVEIEKVVLHPNYSQVDIGLIKLKQKVSVNERVMP
ICLPSKDYAEVGRVGYVSGWGRNANFKFTDHLKYVMLPVADQDQCIRHYEGSTVPEKKTPKSPVGV
QPILNEHTFCAGMSKYQEDTCYGDAGSAFAVHDLEEDTWYATGILSFDKSCAVAEYGVYVKVTSIQ
DWVQKTIAEN

2. Complement component C8 beta chain – P07358

MKNSRTWAWRAPVELFLLCAALGCLSLPGSRGERPHSFGSNAVNKSFAKSRQMRSVDVTLMPIDCE
LSSWSSWTTCDPCQKKRYRYAYLLQPSQFHGEPCNFSDKEVEDCVTNRPCRSQVRCEGFVCAQTGR
CVNRRLLCNGDNDCGDQSDEANCRRIYKKCQHEMDQYWGIGSLASGINLFTNSFEGPVLDHRYYAG
GCSPHYILNTRFRKPYNVESYTPQTQGKYEFILKEYESYSDFERNVTEKMASKSGFSFGFKIPGIFELGI
SSQSDRGKHYIRRTKRFSHTKSVFLHARSDLEVAHYKLKPRSLMLHYEFLQRVKRLPLEYSYGEYRD
LFRDFGTHYITEAVLGGIYEYTLVMNKEAMERGDYTLNNVHACAKNDFKIGGAIEEVYVSLGVSVG
KCRGILNEIKDRNKRDTMVEDLVVLVRGGASEHITTLAYQELPTADLMQEWGDAVQYNPAIIKVKVE
PLYELVTATDFAYSSTVRQNMKQALEEFQKEVSSCHCAPCQGNGVPVLKGSRCDCICPVGSQGLACE
VSYRKNTPIDGKWNCWSNWSSCSGRRKTRQRQCNNPPPQNGGSPCSGPASETLDCS

3. Serum albumin OS-69.3kdw – P02768

MKWVTFISLLFLFSSAYSRGVFRRDAHKSEVAHRFKDLGEENFKALVLIAFAQYLQQCPFEDHVKLV
NEVTEFAKTCVADESAENCDKSLHTLFGDKLCTVATLRETYGEMADCCAKQEPERNECFLQHKDDN
PNLPRLVRPEVDVMCTAFHDNEETFLKKYLYEIARRHPYFYAPELLFFAKRYKAAFTECCQAADKAA
CLLPKLDELRDEGKASSAKQRLKCASLQKFGERAFKAWAVARLSQRFPKAEFAEVSKLVTDLTKVH
TECCHGDLLECADDRADLAKYICENQDSISSKLKECCEKPLLEKSHCIAEVENDEMPADLPSLAADFV
ESKDVCKNYAEAKDVFLGMFLYEYARRHPDYSVVLLLRLAKTYETTLEKCCAAADPHECYAKVFDE
FKPLVEEPQNLIKQNCELFEQLGEYKFQNALLVRYTKKVPQVSTPTLVEVSRNLGKVGSKCCKHPEA
KRMPCAEDYLSVVLNQLCVLHEKTPVSDRVTKCCTESLVNRRPCFSALEVDETYVPKEFNAETFTFH
ADICTLSEKERQIKKQTALVELVKHKPKATKEQLKAVMDDFAAFVEKCCKADDKETCFAEEGKKLV
AASQAALGL

4. Alpha-1-antichymo trypsin - P01011

MERMLPLLALGLLAAGFCPAVLCHPNSPLDEENLTQENQDRGTHVDLGLASANVDFAFSLYKQLVL
KAPDKNVIFSPLSISTALAFLSLGAHNTTLTEILKGLKFNLTETSEAEIHQSFQHLLRTLNQSSDELQLS
MGNAMFVKEQLSLLDRFTEDAKRLYGSEAFATDFQDSAAAKKLINDYVKNGTRGKITDLIKDLDSQT
MMVLVNYIFFKAKWEMPFDPQDTHQSRFYLSKKKWVMVPMMSLHHLTIPYFRDEELSCTVVELKYT

GNASALFILPDQDKMEEVEAMLLPETLKRWRDSLEFREIGELYLPKFSISRDYNLNDILLQLGIEEAFTS KADLSGITGARNLAVSQVVHKAVLDVFEEGTEASAATAVKITLLSALVETRTIVRFNRPFLMIIVPTDT QNIFFMSKVTNPKQA

5. CD5 antigen - O43866

MALLFSLILAICTRPGFLASPSGVRLVGGLHRCEGRVEVEQKGQWGTVCDDGWDIKDVAVLCRELGC GAASGTPSGILYEPPAEKEQKVLIQSVSCTGTEDTLAQCEQEEVYDCSHDEDAGASCENPESSFSPVPE GVRLADGPGHCKGRVEVKHQNQWYTVCQTGWSLRAAKVVCRQLGCGRAVLTQKRCNKHAYGRK PIWLSQMSCSGREATLQDCPSGPWGKNTCNHDEDTWVECEDPFDLRLVGGDNLCSGRLEVLHKGV WGSVCDDNWGEKEDQVVCKQLGCGKSLSPSFRDRKCYGPGVGRIWLDNVRCSGEEQSLEQCQHRF WGFHDCTHQEDVAVICSG

<u>Protein band-p17</u>

1. C-creative protein – P02741

MEKLLCFLVLTSLSHAFGQTDMSRKAFVFPKESDTSYVSLKAPLTKPLKAFTVCLHFYTELSSTRGYSI FSYATKRQDNEILIFWSKDIGYSFTVGGSEILFEVPEVTVAPVHICTSWESASGIVEFWVDGKPRVRKS LKKGYTVGAEASIILGQEQDSFGGNFEGSQSLVGDIGNVNMWDFVLSPDEINTIYLGGPFSPNVLNWR ALKYEVQGEVFTKPQLWP

2. Apolipoprotein A-I OS-30.8kdw – P55056

MKAAVLTLAVLFLTGSQARHFWQQDEPPQSPWDRVKDLATVYVDVLKDSGRDYVSQFEGSALGKQ LNLKLLDNWDSVTSTFSKLREQLGPVTQEFWDNLEKETEGLRQEMSKDLEEVKAKVQPYLDDFQKK WQEEMELYRQKVEPLRAELQEGARQKLHELQEKLSPLGEEMRDRARAHVDALRTHLAPYSDELRQR LAARLEALKENGGARLAEYHAKATEHLSTLSEKAKPALEDLRQGLLPVLESFKVSFLSALEEYTKKL NTQ

3. Alpha-1-antitrypsin OS – PO1009

Isoform 1

MPSSVSWGILLLAGLCCLVPVSLAEDPQGDAAQKTDTSHHDQDHPTFNKITPNLAEFAFSLYRQLAH QSNSTNIFFSPVSIATAFAMLSLGTKADTHDEILEGLNFNLTEIPEAQIHEGFQELLRTLNQPDSQLQLTT GNGLFLSEGLKLVDKFLEDVKKLYHSEAFTVNFGDTEEAKKQINDYVEKGTQGKIVDLVKELDRDTV FALVNYIFFKGKWERPFEVKDTEEEDFHVDQVTTVKVPMMKRLGMFNIQHCKKLSSWVLLMKYLG NATAIFFLPDEGKLQHLENELTHDIITKFLENEDRRSASLHLPKLSITGTYDLKSVLGQLGITKVFSNGA DLSGVTEEAPLKLSKAVHKAVLTIDEKGTEAAGAMFLEAIPMSIPPEVKFNKPFVFLMIEQNTKSPLFM GKVVNPTQK

Isoform 2

MPSSVSWGILLLAGLCCLVPVSLAEDPQGDAAQKTDTSHHDQDHPTFNKITPNLAEFAFSLYRQLAH QSNSTNIFFSPVSIATAFAMLSLGTKADTHDEILEGLNFNLTEIPEAQIHEGFQELLRTLNQPDSQLQLTT GNGLFLSEGLKLVDKFLEDVKKLYHSEAFTVNFGDTEEAKKQINDYVEKGTQGKIVDLVKELDRDTV FALVNYIFFKGKWERPFEVKDTEEEDFHVDQVTTVKVPMMKRLGMFNIQHCKKLSSWVLLMKYLG NATAIFFLPDEGKLQHLENELTHDIITKFLENEDRRSASLHLPKLSITGTYDLKSVLGQLGITKVFSNGA DLSGVTEEAPLKLSKVRSP

4. Complement component C8 gamma chain, P07360

MLPPGTATLLTLLLAAGSLGQKPQRPRRPASPISTIQPKANFDAQQFAGTWLLVAVGSACRFLQEQGH RAEATTLHVAPQGTAMAVSTFRKLDGICWQVRQLYGDTGVLGRFLLQARDARGAVHVVVAETDYQ SFAVLYLERAGQLSVKLYARSLPVSDSVLSGFEQRVQEAHLTEDQIFYFPKYGFCEAADQFHVLDEVR R

5. Immunoglobulin J chain, P01591

MKNHLLFWGVLAVFIKAVHVKAQEDERIVLVDNKCKCARITSRIIRSSEDPNEDIVERNIRIIVPLNNRE NISDPTSPLRTRFVYHLSDLCKKCDPTEVELDNQIVTATQSNICDEDSATETCYTYDRNKCYTAVVPL VYGGETKMVETALTPDACYPD

6. Retinoic acid receptor responder protein – P02753

MRRLLIPLALWLGAVGVGVAELTEAQRRGLQVALEEFHKHPPVQWAFQETSVESAVDTPFPAGIFVR LEFKLQQTSCRKRDWKKPECKVRPNGRKRKCLACIKLGSEDKVLGRLVHCPIETQVLREAEEHQETQ CLRVQRAGEDPHSFYFPGQFAFSKALPRS

7. Retinol-binding protein 4 – Q99969
MKWVWALLLLAALGSGRAERDCRVSSFRVKENFDKARFSGTWYAMAKKDPEGLFLQDNIVAEFSV
DETGQMSATAKGRVRLLNNWDVCADMVGTFTDTEDPAKFKMKYWGVASFLQKGNDDHWIVDTD
YDTYAVQYSCRLLNLDGTCADSYSFVFSRDPNGLPPEAQKIVRQRQEELCLARQYRLIVHNGYCDGR
SERNLL

Protein band-p14

1. Transthyretin-1 – P02766

MASHRLLLLCLAGLVFVSEAGPTGTGESKCPLMVKVLDAVRGSPAINVAVHVFRKAADDTWEPFAS
GKTSESGELHGLTTEEEFVEGIYKVEIDTKSYWKALGISPFHEHAEVVFTANDSGPRRYTIAALLSPYS
YSTTAVVTNPKE

2. Serum amyloid – P35542

MRLFTGIVFCSLVMGVTSESWRSFFKEALQGVGDMGRAYWDIMISNHQNSNRYLYARGNYDAAQR
GPGGVWAAKLISRSRVYLQGLIDCYLFGNSSTVLEDSKSNEKAEEWGRSGKDPDRFRPDGLPKKY

3. Apolipoprotein C – P55056

MSLLRNRLQALPALCLCVLVLACIGACQPEAQEGTLSPPPKLKMSRWSLVRGRMKELLETVVNRTRD
GWQWFWSPSTFRGFMQTYYDDHLRDLGPLTKAWFLESKDSLLKKTHSLCPRLVCGDKDQG

4. Immunoglobulin kappa constant - P01834

RTVAAPSVFIFPPSDEQLKSGTASVVCLLNNFYPREAKVQWKVDNALQSGNSQESVTEQDSKDSTYS
LSSTLTLSKADYEKHKVYACEVTHQGLSSPVTKSFNRGEC

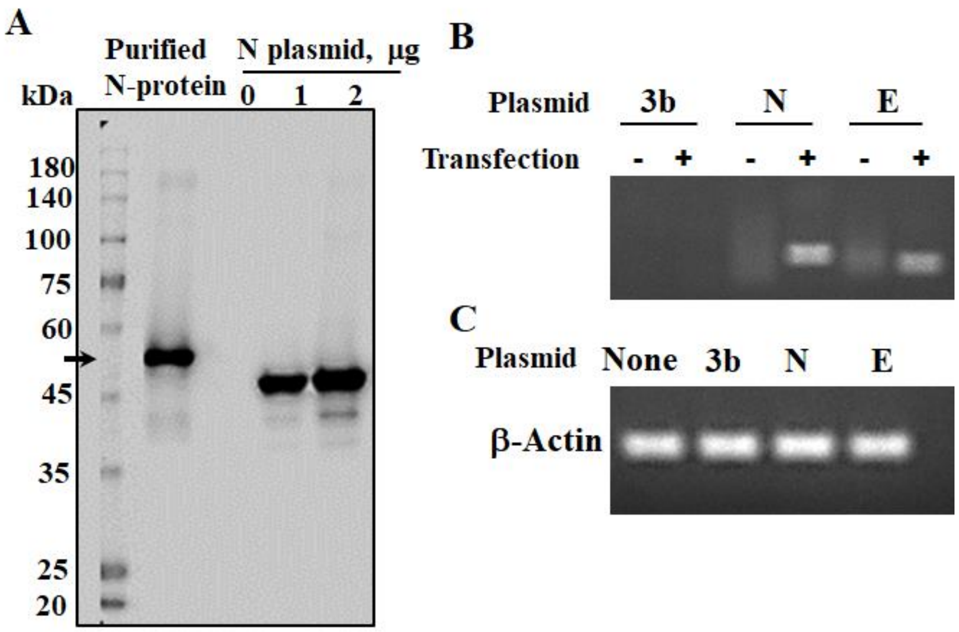

Fig. S2: Cells expression of SARS-CoV-2 proteins
(**A**) SDS-PAGE and Western blot of purified SARS-CoV-2 N-protein C-terminal DYKDDDDK tagged (indicated by an arrow) (Cat. No. S014660-05; Synbio Technologies; NJ, USA) and lysates of SHSY-5Y cells 48h post transfection with 1 or 2 µg of N-Protein encoding plasmid. (**B,C**) SHSY-5Y cells were transfected with empty plasmid or encoding for N, E or 3b proteins. 48h post transfection cells were harvested, and lysates were subjected to PCR using specific primers for 3b, N, E (**B**) or for β-Actin shown as a loading control (**C**).

References

[1] S.N. Naryzny, O.K. Legina, Haptoglobin as a Biomarker, Biochem Mosc-Suppl S 15(3) (2021) 184-198.
[2] I.K. Quaye, Haptoglobin, inflammation and disease, T Roy Soc Trop Med H 102(8) (2008) 735-742.
[3] M.S. Arredouani, A. Kasran, J.A. Vanoirbeek, F.G. Berger, H. Baumann, J.L. Ceuppens, Haptoglobin dampens endotoxin-induced inflammatory effects both in vitro and in vivo, Immunology 114(2) (2005) 263-71.
[4] P. Trayhurn, I.S. Wood, Adipokines: inflammation and the pleiotropic role of white adipose tissue, Br J Nutr 92(3) (2004) 347-55.
[5] J.R. Delanghe, M.L. De Buyzere, M.M. Speeckaert, M.R. Langlois, Haptoglobin phenotype and Parkinson disease risk, Parkinsonism Relat D 22 (2016) 108-109.
[6] C.L. Brannen, J.M. Sodetz, Incorporation of human complement C8 into the membrane attack complex is mediated by a binding site located within the C8beta MACPF domain, Mol Immunol 44(5) (2007) 960-5.
[7] C.L. Parker, J.M. Sodetz, Role of the human C8 subunits in complement-mediated bacterial killing: evidence that C8 gamma is not essential, Mol Immunol 39(7-8) (2002) 453-8.
[8] Y.X. Jin, W.D. Wang, Q.Y. Wang, Y.Y. Zhang, K.R. Zahid, U. Raza, Y.S. Gong, Alpha-1-antichymotrypsin as a novel biomarker for diagnosis, prognosis, and therapy prediction in human diseases, Cancer Cell Int 22(1) (2022).
[9] J. van de Wouw, J.A. Joles, Albumin is an interface between blood plasma and cell membrane, and not just a sponge, Clin Kidney J 15(4) (2022) 624-634.
[10] L. Marnell, C. Mold, T.W. Du Clos, C-reactive protein: ligands, receptors and role in inflammation, Clin Immunol 117(2) (2005) 104-11.
[11] J.D. McFadyen, J. Zeller, L.A. Potempa, G.A. Pietersz, S.U. Eisenhardt, K. Peter, C-Reactive Protein and Its Structural Isoforms: An Evolutionary Conserved Marker and Central Player in Inflammatory Diseases and Beyond, Subcell Biochem 94 (2020) 499-520.
[12] Eppy, Suhendro, L. Nainggolan, C.M. Rumende, The Differences Between Interleukin-6 and C-reactive Protein Levels Among Adult Patients of Dengue Infection with and without Plasma Leakage, Acta Med Indones 48(1) (2016) 3-9.
[13] N.R. Sproston, J.J. Ashworth, Role of C-Reactive Protein at Sites of Inflammation and Infection, Frontiers in Immunology 9 (2018).
[14] A. Sheriff, S. Kayser, P. Brunner, B. Vogt, C-Reactive Protein Triggers Cell Death in Ischemic Cells, Front Immunol 12 (2021) 630430.
[15] D. Wang, B. Hu, C. Hu, F. Zhu, X. Liu, J. Zhang, B. Wang, H. Xiang, Z. Cheng, Y. Xiong, Y. Zhao, Y. Li, X. Wang, Z. Peng, Clinical Characteristics of 138 Hospitalized Patients With 2019 Novel Coronavirus-Infected Pneumonia in Wuhan, China, JAMA 323(11) (2020) 1061-1069.
[16] N. Chen, M. Zhou, X. Dong, J. Qu, F. Gong, Y. Han, Y. Qiu, J. Wang, Y. Liu, Y. Wei, J. Xia, T. Yu, X. Zhang, L. Zhang, Epidemiological and clinical characteristics of 99 cases of 2019 novel coronavirus pneumonia in Wuhan, China: a descriptive study, Lancet 395(10223) (2020) 507-513.
[17] P. Nazemi, S. Seyedalinaghi, A. Azarnoush, A. Mabadi, A.S. Khaneshan, M. Salehi, Serum C-reactive protein greater than 75 mg/dL as an early available laboratory predictor of severe COVID-19: A systematic review, Immun Inflamm Dis 11(12) (2023).
[18] B.J. Cochran, K.L. Ong, B. Manandhar, K.A. Rye, APOA1: a Protein with Multiple Therapeutic Functions, Curr Atheroscler Rep 23(3) (2021) 11.
[19] J.M. Hunt, R. Tuder, Alpha 1 anti-trypsin: one protein, many functions, Curr Mol Med 12(7) (2012) 827-35.
[20] R.A. Stockley, A.M. Turner, alpha-1-Antitrypsin deficiency: clinical variability, assessment, and treatment, Trends Mol Med 20(2) (2014) 105-15.
[21] C.D. Castro, M.F. Flajnik, Putting J Chain Back on the Map: How Might Its Expression Define Plasma Cell Development?, Journal of Immunology 193(7) (2014) 3248-3255.
[22] M. Ueda, Transthyretin: Its function and amyloid formation, Neurochem Int 155 (2022) 105313.
[23] T.H. Brannagan, 3rd, M. Auer-Grumbach, J.L. Berk, C. Briani, V. Bril, T. Coelho, T. Damy, A. Dispenzieri, B.M. Drachman, N. Fine, H.K. Gaggin, M. Gertz, J.D. Gillmore, E. Gonzalez, M. Hanna, D.R. Hurwitz, S.L. Khella, M.S. Maurer, J. Nativi-Nicolau, K. Olugemo, L.F. Quintana, A.M. Rosen, H.H. Schmidt, J. Shehata, M. Waddington-Cruz, C. Whelan, F.L. Ruberg, ATTR amyloidosis during the COVID-19 pandemic: insights from a global medical roundtable, Orphanet J Rare Dis 16(1) (2021) 204.
[24] M. Vieira, M.J. Saraiva, Transthyretin: a multifaceted protein, Biomol Concepts 5(1) (2014) 45-54.
[25] M.A. Liz, T. Coelho, V. Bellotti, M.I. Fernandez-Arias, P. Mallaina, L. Obici, A Narrative Review of the Role of Transthyretin in Health and Disease, Neurol Ther 9(2) (2020) 395-402.
[26] L. Sun, R.D. Ye, Serum amyloid A1: Structure, function and gene polymorphism, Gene 583(1) (2016) 48-57.

[27] M.C. de Beer, T. Yuan, M.S. Kindy, B.F. Asztalos, P.S. Roheim, F.C. de Beer, Characterization of constitutive human serum amyloid A protein (SAA4) as an apolipoprotein, J Lipid Res 36(3) (1995) 526-34.
[28] A. Wolska, M. Reimund, A.T. Remaley, Apolipoprotein C-II: the re-emergence of a forgotten factor, Curr Opin Lipidol 31(3) (2020) 147-153.
[29] A.A. Kei, T.D. Filippatos, V. Tsimihodimos, M.S. Elisaf, A review of the role of apolipoprotein C-II in lipoprotein metabolism and cardiovascular disease, Metabolism 61(7) (2012) 906-921.
[30] G. Vidarsson, G. Dekkers, T. Rispens, IgG subclasses and allotypes: from structure to effector functions, Front Immunol 5 (2014) 520.
[31] L. Sanjurjo, G. Aran, N. Roher, A.F. Valledor, M.R. Sarrias, AIM/CD5L: a key protein in the control of immune homeostasis and inflammatory disease, J Leukoc Biol 98(2) (2015) 173-84.
[32] N. Oskam, M.A.D. Boer, M.V. Lukassen, P.O.D. Heer, T.S. Veth, G. van Mierlo, S.H. Lai, N.I.L. Derksen, V.C. Yin, M. Streutker, V. Franc, M. Siborova, M.J.A. Damen, D. Kos, A. Barendregt, A. Bondt, J.B.V. Goudoever, C.J.C.D. Haas, P.C. Aerts, R.M. Muts, S.H.M. Rooijakkers, G. Vidarsson, T. Rispens, A.J.R. Heck, CD5L is a canonical component of circulatory IgM, P Natl Acad Sci USA 120(50) (2023).
[33] L. Sanchez-Moral, N. Rafols, C. Martori, T. Paul, E. Tellez, M.R. Sarrias, Multifaceted Roles of CD5L in Infectious and Sterile Inflammation, Int J Mol Sci 22(8) (2021).
[34] J.S. Steinhoff, A. Lass, M. Schupp, Biological Functions of RBP4 and Its Relevance for Human Diseases, Front Physiol 12 (2021) 659977.
[35] R. Vollenberg, P.R. Tepasse, M. Fobker, A. Husing-Kabar, Significantly Reduced Retinol Binding Protein 4 (RBP4) Levels in Critically Ill COVID-19 Patients, Nutrients 14(10) (2022).
[36] A.S. Budhu, N. Noy, Direct channeling of retinoic acid between cellular retinoic acid-binding protein II and retinoic acid receptor sensitizes mammary carcinoma cells to retinoic acid-induced growth arrest, Molecular and Cellular Biology 22(8) (2002) 2632-2641.
[37] A. Mazumdar, Y. Haddad, V. Milosavljevic, H. Michalkova, R. Guran, S. Bhowmick, A. Moulick, Peptide-Carbon Quantum Dots conjugate, Derived from Human Retinoic Acid Receptor Responder Protein 2, against Antibiotic-Resistant Gram Positive and Gram Negative Pathogenic Bacteria, Nanomaterials (Basel) 10(2) (2020).
[38] A.R. Sarohan, M. Kizil, A.C. Inkaya, S. Mahmud, M. Akram, O. Cen, A novel hypothesis for COVID-19 pathogenesis: Retinol depletion and retinoid signaling disorder, Cell Signal 87 (2021) 110121.
